# Highly‐Polarized Emission Provided by Giant Optical Orientation of Exciton Spins in Lead Halide Perovskite Crystals

**DOI:** 10.1002/advs.202403691

**Published:** 2024-06-17

**Authors:** Nataliia E. Kopteva, Dmitri R. Yakovlev, Eyüp Yalcin, Ilya A. Akimov, Mikhail O. Nestoklon, Mikhail M. Glazov, Mladen Kotur, Dennis Kudlacik, Evgeny A. Zhukov, Erik Kirstein, Oleh Hordiichuk, Dmitry N. Dirin, Maksym V. Kovalenko, Manfred Bayer

**Affiliations:** ^1^ Experimentelle Physik 2 Technische Universität Dortmund 44227 Dortmund Germany; ^2^ Ioffe Institute Russian Academy of Sciences St. Petersburg 194021 Russia; ^3^ Laboratory of Inorganic Chemistry Department of Chemistry and Applied Biosciences ETH Zürich Zürich CH‐8093 Switzerland; ^4^ Laboratory for Thin Films and Photovoltaics Empa‐Swiss Federal Laboratories for Materials Science and Technology Dübendorf CH‐8600 Switzerland

**Keywords:** excitons, lead halide perovskites, magneto‐photoluminescence, optical spin orientation, spintronics, time‐resolved photoluminescence

## Abstract

Quantum technologic and spintronic applications require reliable material platforms that enable significant and long‐living spin polarization of excitations, the ability to manipulate it optically in external fields, and the possibility to implement quantum correlations between spins, i.e., entanglement. Here it is demonstrated that these conditions are met in bulk crystals of lead halide perovskites. A giant optical orientation of 85% of excitons, approaching the ultimate limit of unity, in FA_0.9_Cs_0.1_PbI_2.8_Br_0.2_ crystals is reported. The exciton spin orientation is maintained during the exciton lifetime of 55 ps resulting in high circular polarization of the exciton emission. The optical orientation is robust to detuning of the excitation energy up to 0.3 eV above the exciton resonance and remains larger than 20% up to detunings of 0.9 eV. It evidences pure chiral selection rules and suppressed spin relaxation of electrons and holes, even with large kinetic energies. The exciton and electron–hole recombinations are distinguished by means of the spin dynamics detected via coherent spin quantum beats in magnetic field. Further, electron–hole spin correlations are demonstrated through linear polarization beats after circularly polarized excitation. These findings are supported by atomistic calculations. All‐in‐all, the results establish lead halide perovskite semiconductors as suitable platform for quantum technologies.

## Graphical abstract

A giant optical spin orientation of 85% of excitons is demonstrated, approaching the ultimate limit of unity, in lead halide FA_0.9_Cs_0.1_PbI_2.8_Br_0.2_ perovskite crystals. The exciton spin orientation is maintained during the exciton lifetime of 55 ps. It is robust to the detuning of the excitation energy up to 0.3 eV above the exciton. It evidences pure chiral selection rules and suppressed spin relaxation of electrons and holes, even with large kinetic energies.

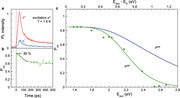

## 1 Introduction

Lead halide perovskite semiconductors are known for their exceptional photovoltaic efficiencies^[^ ^1^, ^2^ ^]^ and optoelectronic properties.^[^ ^3^, ^4^ ^]^ Their simple fabrication technology makes them attractive for applications as solar cells or light emitters. They also demonstrate remarkable spin features, facilitating quantum technologic and spintronic applications.^[^ ^4^, ^5^, ^6^, ^7^ ^]^ To date, the spin physics of halide perovskite semiconductors is an emerging research field, which exploits experimental techniques and physical concepts developed for spins in conventional semiconductors.^[^ ^8^ ^]^ Most spin‐dependent optical techniques work well for perovskite crystals, polycrystalline films, 2D materials, and nanocrystals. These comprise: optical orientation,^[^ ^9^, ^10^ ^]^ optical alignment,^[^ ^10^ ^]^ polarized emission in magnetic field,^[^ ^11^, ^12^ ^]^ time‐resolved Faraday/Kerr rotation,^[^ ^13^, ^14^ ^]^ spin‐flip Raman scattering,^[^ ^15^, ^16^ ^]^ and optically‐detected nuclear magnetic resonance.^[^ ^17^ ^]^ Universal dependence of the electron and hole as well as exciton Landè *g*‐factors on the bandgap energy have been established.^[^ ^15^, ^18^ ^]^ The reported spin dynamics cover huge time ranges from a few picoseconds at room temperature^[^ ^9^, ^19^ ^]^ up to tens of nanoseconds for the spin coherence^[^ ^20^ ^]^ and spin dephasing^[^ ^17^ ^]^ and further up to submilliseconds for the longitudinal spin relaxation times^[^ ^21^ ^]^ at cryogenic temperatures.

Optical orientation is a fundamental phenomenon in spin physics,^[^ ^8^, ^22^ ^]^ where circularly polarized photons generate spin‐oriented excitons and charge carriers, whose spin polarization can be monitored, also dynamically, via polarized photoluminescence, Faraday/Kerr rotation, spin‐dependent photocurrents, etc. Optical pulses with sub‐picosecond duration can be used for ultrafast spin orientation, manipulation, and readout operations as required for quantum information technologies. For lead halide perovskites, optical orientation using pulsed excitation was used to launch the spin dynamics in polycrystalline films,^[^ ^9^, ^13^ ^]^ bulk crystals,^[^ ^14^, ^17^ ^]^ nanocrystals,^[^ ^19^, ^23^ ^]^ nanoplatelets,^[^ ^24^ ^]^ and 2D materials.^[^ ^25^, ^26^, ^27^ ^]^ All‐optical manipulation of carrier spins in singly‐charged CsPbBr_3_ nanocrystals recently was demonstrated at room temperature.^[^ ^28^ ^]^

The electronic band structure of lead halide perovskites is favorable for optical spin orientation of charge carriers and excitons, as the selection rules for optical transitions allow 100% carrier spin polarization in absorption of circularly polarized photons and also up to 100% polarized luminescence. Note, that in conventional III–V and II–VI bulk semiconductors (like GaAs, CdTe, etc.) the optical orientation measured in emission is limited to 25%. In time‐resolved experiments on polarized differential transmission, excitation of highly polarized carrier spins was demonstrated,^[^ ^9^, ^24^, ^26^ ^]^ but the spin relaxation of carriers in the temperature range 77−300 K is fast (<3 ps) so that the carriers become depolarized during their lifetime. The differential transmission technique does not allow one to distinguish the polarization of electrons, holes, and excitons.

Using continuous‐wave excitation, only small degrees of optical orientation measured via circular polarization of photoluminescence so far were reported for MAPbBr_3_ polycrystalline films (degree of 3.1%^[^ ^5^ ^]^ at 10 K temperature, 2%^[^ ^29^ ^]^ and 8%^[^ ^30^ ^]^ at 77 K), for MAPbI_3_ (0.15%^[^ ^29^ ^]^ at 77 K), and for CsPbI_3_ NCs (4%^[^ ^10^ ^]^ at 2 K). Thus, it has remained a challenge to examine the maximum achievable optical orientation in perovskites, to identify the limiting mechanisms involved in spin generation and relaxation, and to clarify their specifics for electrons and holes, as well as excitons.

In this paper, we focus on the exciton spin dynamics and use several experimental approaches to distinguish it from the carrier spin dynamics. We overcome this challenge by selecting a suitable material FA_0.9_Cs_0.1_PbI_2.8_Br_0.2_ bulk perovskite and applying several complementary experimental approaches to study optical orientation and pinpoint the contribution of excitons. We demonstrate that a very high degree of optical orientation up to 85% can be achieved for excitons in FA_0.9_Cs_0.1_PbI_2.8_Br_0.2_ crystals. It is surprisingly robust with respect to the detuning of the laser excitation energy from the exciton resonance by up to 0.3 eV, evidencing the suppression of the carrier spin relaxation mechanisms typical for conventional III–V and II–VI semiconductors. Atomistic calculations based on density functional theory and empirical tight‐binding models accounting for the spin‐dependent optical matrix elements at large carrier wave vectors support these observations. Time‐resolved photoluminescence allows us to distinguish the excitons with 55 ps lifetime from electron–hole recombination in the spin dynamics, detected via coherent spin beats in magnetic field that are induced by circularly polarized excitation and detected in linear or circular polarization, respectively. The measured linear polarization demonstrates that electron‐hole spin correlations arise in magnetic field.

## 2 Results

For this study, we choose a bulk single crystal of the FA_0.9_Cs_0.1_PbI_2.8_Br_0.2_ hybrid organic–inorganic lead halide perovskite with high structural quality and small inhomogeneous broadening of the exciton resonance. The small additions of Cs and Br to the basic FAPbI_3_ composition allow one to approach the tolerance factor unity. Therefore, this crystal upholds cubic lattice symmetry also at cryogenic temperatures, as confirmed by the isotropic electron and hole *g*‐factors measured at T=1.6 K.^[^ ^15^ ^]^

**Figure 1:**
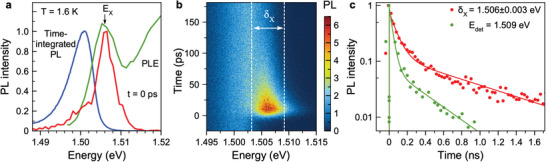
Exciton features in bulk FA_0.9_Cs_0.1_PbI_2.8_Br_0.2_ crystal. a) Time‐integrated photoluminescence spectrum (blue line) excited at $E_{\text{exc}}=1.669$ eV photon energy, using P=10 mW ${\mathrm{cm}}^{-2}$ laser power. T=1.6 K. Photoluminescence excitation spectrum (green line) detected at $E_{\text{det}}=1.496$ eV. $E_{X}$ denotes the exciton resonance. PL spectrum at the moment of excitation t=0 ps for pulsed excitation (red line). b) Contour plot of time‐resolved photoluminescence excited with 200 fs laser pulses. c) Recombination dynamics detected at $E_{\text{det}}=1.509$ eV (green) and integrated over the (1.503−1.509) eV spectral range around the exciton line maximum at $E_{X}=1.506$ eV (red). Lines show bi‐exponential fits with decay times: $\tau_{R1}=55$ ps and $\tau_{R2}=840$ ps for $E_{\text{det}}=1.506$ eV, and $\tau_{R1}=35$ ps and $\tau_{R2}=380$ ps for $E_{\text{det}}=1.509$ eV.

The optical properties of the studied crystal are illustrated in **Figure** **1**a, more details can be found in refs. [15, 17, 18, 31]. At the temperature of T=1.6 K the exciton resonance is seen at 1.506 eV in the photoluminescence excitation (PLE) spectrum. The exciton binding energy should be close to the 14 meV for FAPbI_3_,^[^ ^32^ ^]^ which gives us the bandgap energy of $E_{g}=1.520$ eV in the studied crystal. The time‐integrated photoluminescence (PL) spectrum measured using pulsed excitation shows a line with a maximum at 1.501 eV and a full width at half maximum of 5 meV. The recombination dynamics of this line cover a large temporal range from 700 ps to 44 µs with a large spectral dispersion (Section S3, Supporting Information), indicating a multitude of recombination processes including that of spatially separated charge carriers.^[^ ^17^ ^]^ The coherent spin dynamics of resident electrons and holes following their optical orientation show nanosecond spin dephasing times in such crystals.^[^ ^17^ ^]^

Here, we focus on the spin properties of excitons with short recombination times, for which we use time‐resolved photoluminescence (TRPL) recorded with a streak camera to isolate the exciton signals. The PL dynamics are shown as a color map in Figure 1b. Right after the photogeneration at t=0 ps, the emission has its spectral maximum at 1.506 eV, equal to the exciton resonance in the PLE spectrum at $E_{X}=1.506$ eV (red line in Figure 1a). The spectrally‐integrated exciton emission (red line in Figure 1c) is observable on time scales up to 1.7 ns, showing a double exponential decay. The fast decay time $\tau_{\text{R1}}=55$ ps is assigned to exciton recombination, and the longer one $\tau_{\text{R2}}=840$  ps to recombination of spatially separated electrons and holes. Decay times are evaluated by Equation (7) in Section 4. It is a specific of lead halide perovskites that these processes overlap spectrally, which complicates the interpretation of the recombination and spin dynamics.^[^ ^14^, ^17^ ^]^ The assignment of the $\tau_{\text{R2}}$ time to recombination of separated electrons and holes is confirmed by the results on the coherent spin dynamics in magnetic field measured by time‐resolved Kerr rotation in ref. [17] and in this study by time‐resolved polarized emission, see Figure 4a. The dependences of the exciton and electron–hole pair recombination times on temperature and excitation power are given in Figure S5 (Supporting Information). Note, that the excitation density of 10 mW ${\mathrm{cm}}^{-2}$ corresponds to a relatively low exciton density of 10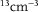, so that the exciton–exciton interaction can be neglected. By the green line in Figure 1c we show the PL dynamics measured at 1.509 eV, i.e., at the high energy wing of the exciton line. The dynamics here having values $\tau_{\text{R1}}=35$ ps and $\tau_{\text{R2}}=380$  ps are faster than the times obtained by integrating over the exciton line, evidencing that energy relaxation of excitons contributes to the spectral dependence of their dynamics. We showed that recently for the FA_0.9_Cs_0.1_PbI_2.8_Br_0.2_ crystals by means of transient photon echo spectroscopy.^[^ ^31^ ^]^

**Figure 2:**
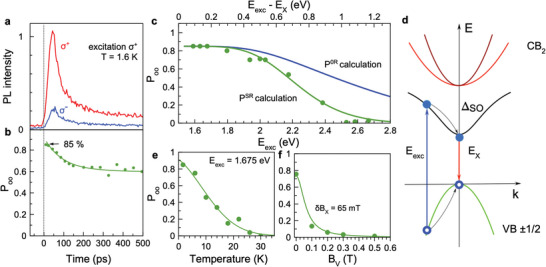
Optical orientation of exciton spins recorded at the exciton energy of 1.506 eV. a) PL dynamics detected in $\sigma^{+}$ (red line) and $\sigma^{-}$ (blue line) polarization for $\sigma^{+}$ excitation at $E_{\text{exc}}=1.669$ eV with P=10 mW ${\mathrm{cm}}^{-2}$. T=1.6 K. b) Dynamics of the optical orientation degree $P_{\mathrm{oo}}\left(t\right)$. Line is an exponential fit between the 0.85 and 0.60 levels yielding the decay time of 100 ps. c) Dependence of $P_{\mathrm{oo}}\left(t=0\right)$ on the excitation energy $E_{\text{exc}}$ (symbols). The upper axis shows the detuning from the exciton resonance $E_{\text{exc}}-E_{X}$. The blue line is the theoretical curve $P^{0R}$ from Figure 5b multiplied by the depolarization factor 0.85 to match the experimental value of $P_{\mathrm{oo}}=0.85$ at small detunings, see Figure S8b (Supporting Information). The green line is the theoretical curve $P^{\mathrm{SR}}$ calculated with accounting for the Elliott–Yafet spin relaxation due to interaction with longitudinal optical phonons, see Figure S8b and discussion in section S8.C.3 (Supporting Information). d) Sketch of the band structure of lead halide perovskites with cubic symmetry. VB and ${\mathrm{CB}}_{1}$ denote the valence and conduction bands with electron and hole spins ±1/2. The ${\mathrm{CB}}_{2}$ band consisting of the heavy (he) and light (le) electron subbands is shifted from ${\mathrm{CB}}_{1}$ by the spin‐orbit splitting $\Delta_{SO}$. e) Temperature dependence of $P_{\mathrm{oo}}\left(t=0\right)$ (symbols). The line is a guide to the eye. f) $P_{\text{oo}}$ dependence on the magnetic field applied in the Voigt geometry ($B_{V}$) for $\sigma^{+}$ excitation at $E_{\text{exc}}=1.675$ eV with P=30 mW ${\mathrm{cm}}^{-2}$. T=1.6 K. Each point is obtained by integration of the PL dynamics over 2 ns. Line is the fit with Equation (S16, Supporting Information), assuming $\delta B_{X}=65$ mT.

**Figure 3:**
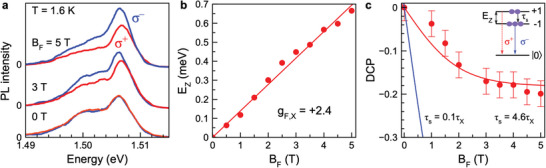
Exciton Zeeman splitting and polarization in Faraday magnetic field at T=1.6 K. a) PL spectra integrated over $\tau_{X}$ for $\sigma^{+}$ (red) and $\sigma^{-}$ (blue) polarization in the longitudinal magnetic field $B_{F}=5T$. The exciting laser is linearly polarized, $E_{\text{exc}}=1.669$ eV with P=10 mW ${\mathrm{cm}}^{-2}$. b) Exciton Zeeman splitting as a function of $B_{F}$ (symbols). The line is $B_{F}$‐linear fit with $g_{\text{F,X}}=+2.4$. c) Degree of circular polarization dependence on $B_{F}$ (red circles). The red line is fit using Equation (5) with T=1.6 K for $\tau_{s}=4.6\tau_{X}$. The blue line is the calculated result for the condition $\tau_{s}=0.1\tau_{X}$ at T=1.6 K. Inset shows schematically the populations of excitons on their Zeeman‐split spin sublevels and the resulting polarized emission. |0⟩ represents the crystal ground state.

**Figure 4:**
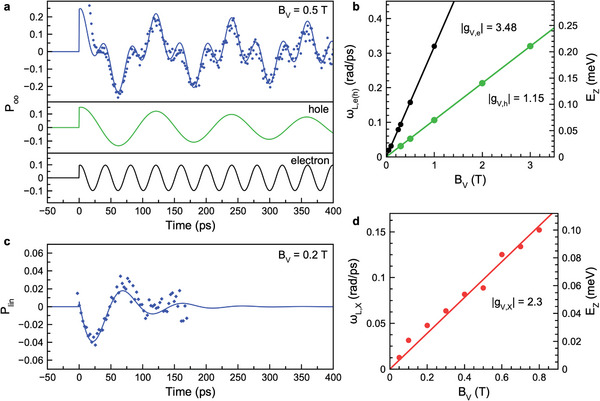
Spin precession of excitons and resident carriers in Voigt magnetic field measured by TRPL at T=1.6 K. a) Dynamics of the optical orientation degree $P_{\mathrm{oo}}\left(t\right)$ measured for $B_{V}=0.5$ T using $\sigma^{+}$ excitation at $E_{\text{exc}}=1.675$ eV with P=30 mW ${\mathrm{cm}}^{-2}$ (symbols). $E_{\text{det}}=E_{X}=1.506$ eV. The blue line is fit with Equation (8) including electron and hole contributions, which are shown by the black and green lines, respectively. b) Dependence of the Larmor precession frequencies of electron (black circles) and hole (green circles) on $B_{V}$. Linear fits give $|g_{\text{V,e}}|=3.48$ and $|g_{\text{V,h}}|=1.15$. c) Dynamics of the linear polarization degree $P_{\text{lin}}\left(t\right)$ measured at $B_{V}=0.2$ T using $\sigma^{+}$ polarized excitation as in panel (a) (the symbols). The line is fit with Equation (9) using $\omega_{L,X}=0.048$ rad/ps and $\tau_{X}=55$ ps. d) Magnetic field dependence of the Larmor precession frequency from $P_{\text{lin}}\left(t\right)$ (the symbols). The line is linear fit with $|g_{\text{V,X}}|=2.3$. Right scale gives the corresponding Zeeman splitting.

The PL dynamics have two contributions from exciton and from electron‐hole pair recombination (see Figure 1c). These contributions spectrally overlap but can be distinguished in the time domain. To concentrate on the spin dynamics of excitons in the polarization‐resolved measurements, we isolate the exciton recombination dynamics in the energy range of $\delta_{X}=1.506\pm 0.003$ eV around the exciton line maximum at $E_{X}=1.506$ eV, using temporally and spectrally resolved PL, see the red line in Figure 1c. These dynamics have decay times of $\tau_{R1}=55$ ps and $\tau_{R2}=840$ ps. At lower spectral energies much longer recombination dynamics up 40 µs are detected, which contribute to the time‐integrated PL and to the low‐energy shoulder at PL right after the excitation. We attribute them to the recombination of spatially separated carriers, see data and discussion in Section S3 (Supporting Information).

### 2.1 Optical Orientation of Exciton Spins

The dynamics of the $\sigma^{+}$ and $\sigma^{-}$ circularly polarized PL after excitation with $\sigma^{+}$ polarized pulses are shown in **Figure** **2**a. The strong difference in their amplitudes in favor of the $\sigma^{+}$ polarized signal demonstrates a large degree of optical orientation defined as:

(1) $$P_{\mathrm{oo}}=\frac{I^{++}-I^{+-}}{I^{++}+I^{+-}}$$

which is plotted as function of time in Figure 2b. Here $I^{++}$ and $I^{+-}$ are the intensities of the $\sigma^{+}$ and $\sigma^{-}$ polarized emission components for $\sigma^{+}$ polarized excitation. Strikingly, the initial value of $P_{\mathrm{oo}}$ reaches 0.85 (85%), dropping during the first 100 ps to a saturation level of 0.60 (60%) followed by almost no further decay. Commonly, the decay of $P_{\mathrm{oo}}\left(t\right)$ is attributed to spin relaxation. In our case, the situation is different as both excitons and electron–hole recombination are contributing to $P_{\mathrm{oo}}$ and the exciton spin relaxation time exceeds the exciton lifetime $\tau_{X}$ (see below and in Section S5, Supporting Information). At the initial stage, mainly excitons contribute to $P_{\mathrm{oo}}\left(t\right)$. After their recombination, the PL signal is dominated by long‐lived carriers with $P_{\mathrm{oo}}=0.60$. Therefore, the $P_{\text{oo}}$ decay from 0.85 to 0.60 is determined by the exciton lifetime. A detailed consideration of optical spin orientation for the electrons and holes is given in ref. [33].

Assuming in agreement with the experiment, that the lifetime and spin relaxation time of electrons and holes exceeds by far the exciton lifetime, we obtain for limiting value of the circular polarization degree^[^ ^22^ ^]^

(2) $$P_{\text{oo}}\left(t\gg \tau_{X},\tau_{s}\right)=\frac{\tau_{s}}{\tau_{X}+\tau_{s}}P_{\mathrm{oo}}\left(0\right)$$

Here $\tau_{s}$ is the exciton spin relaxation time, and $\tau_{X}$ is the exciton lifetime. The initial polarization value, $P_{\mathrm{oo}}\left(0\right)$, is limited by the maximum $P_{\text{oo}}^{\max}=1$ (100%), dictated by the band structure for optical transitions at the absorption edge. Taking $\tau_{X}=\tau_{\text{R1}}=55$ ps and $P_{\text{oo}}=0.85$ from experiment, we obtain $\tau_{s}=220$ ps.

Surprisingly, such a high degree of initial optical orientation is also measured for a considerable detuning of the laser energy from the exciton resonance by $E_{\text{exc}}-E_{X}=0.163$ eV. We attribute this result to the strict selection rules for optical transition between the involved bands with low mixing and the absence of the Dyakonov–Perel mechanism in the cubic crystal phase of the perovskite material. By contrast, in conventional III–V and II–VI semiconductors with zinc‐blend crystal structure a high $P_{\text{oo}}$ can be detected only for resonant or close‐to‐resonant excitation due to:^[^ ^8^, ^22^, ^34^ ^]^ i) the reduction of the pure selection rules caused by band mixing and ii) the efficient Dyakonov–Perel mechanism resulting in accelerated spin relaxation of the charge carries large kinetic energies. The dependence of the initial spin polarization of excitons on their excess energy in ${\mathrm{CsPbBr}}_{3}$ nanoplatelets was reported in ref. [24] and assigned to spin relaxation effects. At low temperatures, however, the size quantization of electrons and holes in nanoplatelets results in drastically different spin relaxation processes unrelated to the spin‐orbit interaction,^[^ ^8^, ^34^ ^]^ while at high temperatures the relaxation is governed by the charge carrier interaction with phonons.^[^ ^35^ ^]^

In Figure 2c, $P_{\mathrm{oo}}\left(0\right)$ is shown for a large range of excitation energy detunings from 0.08 up to 1.2 eV. The value of 0.85 is preserved up to the detuning of 0.3 eV and then it smoothly decreases, approaching zero at 1.05 eV. These detunings exceed by far the exciton binding energy so that the photogenerated electron and hole quickly separate in space because of their opposite momenta. Thus, the direct formation of excitons from photogenerated electron–hole pairs is unlikely. Hence, the carriers relax within a few picoseconds to the bottom of the conduction and the top of the valence bands and, at cryogenic temperatures, can either form excitons or become localized, from where they can recombine as spatially separated carriers. We stress that the large value of $P_{\text{oo}}$ for the excitons indicates that: i) the chiral selection rules are fulfilled not only at the absorption edge, but even for large detunings, ii) the carriers do not lose their spin polarization during energy relaxation, and iii) when bound to an exciton in vicinity of the bandgap, they show almost negligible spin relaxation within the exciton lifetime at T=1.6 K. Clean chiral selection rules and the absence of efficient spin flips of charge carriers are confirmed by our atomistic modeling, see Section. “Theoretical analysis” below and Section S8 (Supporting Information). We also note that the considerable optical orientation implies, in addition to pure selection rules, suppressed spin relaxation. It provides indirect evidence of the presence of an inversion center in our sample: Otherwise, the momentum‐dependent spin‐orbit splitting related to the inversion asymmetry provides efficient spin depolarization via the Dyakonov–Perel mechanism.^[^ ^8^, ^35^ ^]^

We have checked that the depolarization in the transverse magnetic field via the Hanle effect, see Figure 2f, gives a similar exciton spin relaxation time of 300 ps (Section S5C, Supporting Information). The huge value of $P_{\mathrm{oo}}=0.6$ after exciton recombination, Figure 2b, demonstrates a remarkably long‐lasting free carrier spin relaxation. The estimates based on the Hanle effect model give $\tau_{s,e/h}\approx 1200$ ps (Section S5C, Supporting Information).

We find a strong temperature dependence of $P_{\text{oo}}$ (Figure 2e) with vanishing polarization for temperatures exceeding 30 K. We attribute that to thermally activated spin relaxation for excitons and free carriers and their interaction with phonons.^[^ ^17^, ^35^, ^36^ ^]^

### 2.2 Polarization of Bright Excitons in Longitudinal Magnetic Field

In order to address the spin dynamics of the excitons in their ground state and separate it from the spin dynamics of carriers at larger energies, we analyze the degree of circular polarization (DCP) induced by a magnetic field applied in the Faraday geometry, $P_{c}\left(B_{F}\right)$, using linearly polarized excitation. Polarized PL spectra integrated over the exciton lifetime are shown in **Figure** **3**a. The exciton line at 1.506 eV demonstrates Zeeman splitting of the bright (optically active) exciton states with angular momentum z‐components $J_{z}=\pm 1$:

(3) $$E_{Z}=\mu_{B}g_{X}B$$

where $g_{X}$ is the exciton *g*‐factor and $\mu_{B}$ is the Bohr magneton (see also inset in Figure 3c). The magnetic field dependence of the Zeeman splitting (Figure 3b) gives us $g_{\text{F,X}}=+2.4$, which coincides well with the known values for FA_0.9_Cs_0.1_PbI_2.8_Br_0.2_.^[^ ^18^ ^]^ The positive sign of $g_{X}$ corresponds to the lower‐energy sublevel with $\sigma^{-}$ polarization, see the inset in Figure 3c. A detailed study of the bright exciton *g*‐factors in lead halide perovskites is performed in ref. [18] by the magneto‐reflectivity method. The exciton *g*‐factor is isotropic and approximately independent of the bandgap energy in a large range from 1.5 to 3.2 eV.

Figure 3a shows that the PL emission becomes circularly polarized in a magnetic field with stronger emission in $\sigma^{-}$ polarization for $B_{F}>0$, evidencing the stronger population of the lower energy Zeeman sublevel, see the inset of Figure 3c. The DCP is evaluated as^[^ ^22^ ^]^

(4) $$P_{c}\left(B_{F}\right)=\frac{I^{+}-I^{-}}{I^{+}+I^{-}}$$

where $I^{+}$ and $I^{-}$ are the intensities of the $\sigma^{+}$ and $\sigma^{-}$ polarized emission components. The $P_{c}$ dependence on $B_{F}$ is shown in Figure 3c. The DCP magnitude increases linearly in small magnetic fields and approaches saturation at $P_{c}=-0.20$ at $B_{F}=5$ T. This behavior is typical for excitons undergoing thermalization between the Zeeman levels and can be described by:

(5) $$P_{c}\left(B_{F}\right)=-\frac{\tau_{X}}{\tau_{X}+\tau_{s}}\tanh\left(\frac{g_{\text{F,X}}\mu_{B}B_{F}}{2k_{B}T}\right)$$

We fit the experimental data with this equation using T=1.6 K and $\tau_{X}=55$ ps, see the red line in Figure 3c. It gives $\tau_{s}=4.6\tau_{X}=250$ ps as the only fit parameter, which is close to the $\tau_{s}=220$ ps extracted from optical orientation. Interestingly, the long spin relaxation time compared to the lifetime results in opposite trends of DCP and optical orientation: i) it significantly reduces the DCP, as compared to the fully thermalized case ($\tau_{s}\ll \tau_{X}$) shown by the blue line in Figure 3c, and ii) it results in high values of optical orientation, see Equation (2).

### 2.3 Spin Precession in Transverse Magnetic Field

The information on the spin dynamics can be enriched by the application of a magnetic field in the Voigt geometry, perpendicular to the light k‐vector ($B_{V}⊥k$). In this case, the exciton and/or carrier spins, which have been optically‐oriented along the k‐vector, undergo Larmor precession about the field direction with the frequency:

(6) $$\omega_{L}=g\mu_{B}B_{V}/\hbar$$

ℏ is the Plank constant. The spin dynamics measured thereby provide access to the *g*‐factor value and to the spin dephasing time $T_{2}^{*}$, the latter obtained from the signal decay. TRPL can be used to directly monitor the spin precession quantum beats of excitons and charge carriers via the circular and linear polarization degree of emission.^[^ ^37^, ^38^, ^39^ ^]^

**Figure** **4**a shows the dynamics of $P_{\mathrm{oo}}\left(t\right)$ measured for $B_{V}=0.5$ T at the exciton energy ($E_{\text{det}}=E_{X}=1.506$ eV). A complex pattern of spin beats is observed with a weak decay within the temporal range of 400 ps. The corresponding decay time greatly exceeds the exciton lifetime, so we assign the signal to the coherent spin precession of resident carriers. The signal contains two oscillating components with Larmor frequencies corresponding to the *g*‐factors of the electron ($|g_{\text{V,e}}|=3.48$) and the hole ($|g_{\text{V,h}}|=1.15$), see the fits in Figure 4a and the magnetic field dependences of the Larmor frequencies in Figure 4b, in agreement with the time‐resolved Faraday/Kerr rotation measurements on the same perovskite crystal.^[^ ^17^ ^]^ The absence of any offset in the Zeeman splittings for $B_{V}\to 0$ confirms that the signal arises from pairs of spatially separated electrons and holes whose exchange interaction is negligible. The dependence $P_{\mathrm{oo}}\left(t\right)$ is accurately described by the model approach developed in Section S7 (Supporting Information) for the case of zero splitting between the singlet and triplet exciton states ($\Delta_{X}=0$), Figure S6b.

Strikingly, in the Voigt geometry the exciton PL for circularly polarized excitation becomes linearly polarized with a degree defined as $P_{\text{lin}}=\left(I^{+⊥}-I^{+∥}\right)/\left(I^{+⊥}+I^{+∥}\right)$. Here, $I^{+⊥}$ and $I^{+∥}$ are the PL intensities in the linear polarizations perpendicular and parallel to the magnetic field direction. The dynamics of $P_{\text{lin}}\left(t\right)$ measured at $B_{V}=0.2$ T are shown in Figure 4c. The polarization degree decays with the time $\tau_{X}=55$ ps, during which it precesses with the Larmor frequency corresponding to $|g_{\text{V,X}}|=2.3$ (Figure 4d), close to the exciton *g*‐factor measured from PL, $g_{\text{F,X}}=+2.4$ (Figure 3b) and to the sum of the carrier *g*‐factors $g_{\text{V,e}}+g_{\text{V,h}}=+2.33$. These facts allow us to reliably assign the spin beats detected in the linear polarization degree to the dynamics of the bright exciton states with $J_{z}=\pm 1$ having a finite exchange interaction ($\Delta_{X}>0$), see the Figure S6 (Supporting Information). These linear polarization beats are a clear indication of electron and hole spin correlations. Indeed, individual charge carrier spin polarization can produce only circular polarization of emission, while linear polarization $P_{\mathrm{lin}}$ is governed by the quantum mechanical average $\left\langle {\hat{s}}_{x}^{e}{\hat{s}}_{x}^{h}-{\hat{s}}_{y}^{e}{\hat{s}}_{y}^{h}\right\rangle$ with ${\hat{s}}^{e/h}$ being the electron and hole spin operators and x,y labeling their in‐plane components, see Section S7B (Supporting Information). Thus, for non‐zero $P_{\mathrm{lin}}$ a non‐zero average of ${\hat{s}}_{x}^{e}{\hat{s}}_{x}^{h}$ or ${\hat{s}}_{y}^{e}{\hat{s}}_{y}^{h}$ should be present, for which case electron–hole spin correlation is required. Spin precession in magnetic field results in oscillations of $P_{\mathrm{lin}}$. Hence, the polarization of both electron and hole spins is needed to obtain linear polarization, in stark contrast to the case of circular polarization which can appear as a result of the recombination of a polarized carrier with an unpolarized one. The presence of correlations is important for the generation of entangled electron–hole spin states, see Section S7B (Supporting Information) for details.

We stress that the beats both in circular and in linear polarization can be excited highly nonresonantly, for example, with a detuning $E_{\text{exc}}-E_{X}=0.17$ eV.

### 2.4 Theoretical Analysis

Bulk perovskites are strongly different from conventional III–V and II–VI semiconductors,^[^ ^8^, ^22^ ^]^ owing to the presence of an inversion center in the point symmetry group and an “inverted” band structure with simple, twofold degenerate conduction and valence bands at the R point of the Brillouin zone, see Figure 2d. The former nullifies the spin‐orbit splitting of the bands and suppresses the spin relaxation of charge carriers due to the absence of the Dyakonov–Perel' spin relaxation mechanism, and the spin relaxation is basically governed by rather inefficient Elliott–Yafet mechanism and also the electron–hole exchange interaction in the exciton. The latter allows for 100% optical orientation of charge carriers and excitons in the interband transitions. The measured high degree of optical orientation and its robustness against excitation detuning are in agreement with these qualitative considerations. However, the loss of optical orientation for excitation with a detuning $E_{\text{exc}}-E_{X}>0.6$ eV (Figure 2c) calls for a detailed theoretical analysis. Note, that variation of selection rules due to k·p‐conduction and valence band mixing described within the Kane model and due to the $k^{2}$ mixing between the spin‐orbit split conduction bands described by the Luttinger Hamiltonian, generally, require higher detunings $E_{\text{exc}}-E_{X}≳E_{g},\Delta$ (Δ is the conduction band spin‐orbit splitting), see refs. [40, 41] for estimates. At such detunings a simplified few band k·p model is inapplicable that calls for a microscopic analysis presented below.

The general scheme of optical orientation calculations includes three basic steps: 1) A circularly polarized optical pulse generates electrons and holes with wave vector $k_{0}$ satisfying the momentum and energy conservation laws: the momentum is equal for both carriers and their energy is equal to the incident photon energy (here we neglect the momentum of the incident photon). For a fixed photon energy, there is a set of possible $k_{0}$. The spin states of the photoexcited carriers are defined by the polarization of the incident light and can be described by the density matrix of the carriers ${\hat{\rho }}^{c,v}\left(k_{0}\right)$. 2) The electrons and holes lose their kinetic energy mostly due to interaction with optical phonons. Assuming a concrete mechanism of energy relaxation, one may compute the density matrix of the carriers at the R point ${\hat{\rho }}^{c,v}\left(k_{R}\right)$. 3) The electrons and holes recombine at the R point.

**Figure 5:**
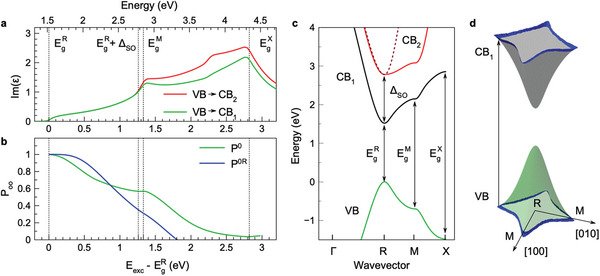
Calculation of optical orientation in lead halide perovskites. a) Imaginary part of the dielectric function calculated in the empirical tight‐binding method for the $\mathrm{VB}\to {\mathrm{CB}}_{1}$ (green) and $\mathrm{VB}\to {\mathrm{CB}}_{2}$ (red) transitions, using the parameters from ref. [42] corrected for the experimental data, see Section S8 (Supporting Information). Vertical lines show the energies of the transitions at the R, M, and X points. The upper axis is the energy detuning from the bandgap energy at the R point ($E_{g}^{R}$). b) Dependence of the optical orientation degree on the excitation energy detuning from $E_{g}^{R}$, $E_{\text{exc}}-E_{g}^{R}$, calculated within the two models explained in the text. Vertical lines show the energies of the R, M, and X points. c) Band diagram of the bulk FA_0.9_Cs_0.1_PbI_2.8_Br_0.2_ crystal along the Γ→R→M→X path. d) Illustration of the wave vectors $k_{0}$ in the (001) plane contributing to the transitions with energy close to $E_{g}^{M}$. One can see the large warping of the band structure leading to electron/hole distributions highly anisotropic in the k‐space.

We start the theoretical analysis by calculating the absorption. We use the tight‐binding approach with the parameters of ${\mathrm{CsPbI}}_{3}$ from ref. [42], corrected for the measured bandgap, see Section S8B (Supporting Information). For the detailed discussion of k·p analysis of tight‐binding calculations, we refer to Supporting Information of refs. [15, 43]. The imaginary part of the dielectric function without excitonic effects is presented in **Figure** **5**a. In agreement with previous calculations,^[^ ^44^, ^45^ ^]^ the absorption increases from the bandgap at the R point up to the M point, see Figure 5c for the band dispersion.

Figure 5b shows the optical orientation degree calculated in the two models described in Section S8 (Supporting Information): $P^{0R}$ corresponds to the “effective phonon” model, where the direct transition from $k_{0}$ to the R point is assumed to occur via spin‐independent electron/hole‐phonon interaction, and $P^{0}$ corresponds to the “effective emission” model where we calculate the polarization of the emission assuming recombination in the excited states. The band energies, matrix elements of velocity, and amplitudes of the transitions between $k_{0}$ and $k_{R}$ are calculated in the empirical tight‐binding approach (Section S6, Supporting Information). As can be seen in Figures 5b and 2c, the calculated $P_{\mathrm{oo}}\left(E_{\mathrm{exc}}\right)$ dependence qualitatively follows the experimental data: it decreases starting from the detuning of ≈0.3 eV, but remains large <0.4 up to detunings of ≈1.0 eV. From the behavior of $P^{0}\left(E_{\mathrm{exc}}\right)$ we conclude that the depolarization to a large extent originates from deviations of the selection rules from the strict ones away from the R point. For even higher detunings ≳1.3 eV, the transitions to the spin split‐off conduction band, ${\mathrm{CB}}_{2}$, set in which further reduce the polarization (not included in the calculations). Additional depolarization results from the spin‐flip scattering of charge carriers during their energy relaxation, see the $P^{\mathrm{SR}}\left(E_{\mathrm{exc}}\right)$ dependence in Figure 2c. We note that the electron–hole exchange interaction is inefficient since: i) for electrons and hole having large kinetic energies the phonon emission rates exceed by far the electron–hole scattering rate, and ii) for excitons in vicinity of the bandgap their short lifetime prevents a significant depolarization by the exchange interaction.

## 3 Conclusion

In conclusion, we have used time‐ and polarization‐resolved photoluminescence to demonstrate a giant, unprecedentedly high optical orientation degree up to 85% in bulk perovskite crystals. The orientation degree is amazingly robust with respect to a significant, up to 0.3 eV, detuning of the excitation energy away from the fundamental absorption edge and is fully suppressed only for detunings exceeding 1.0 eV. The combination of symmetry analysis and atomistic calculations shows that the remarkable optical orientation is a consequence of the unique properties of lead halide perovskites, namely the “clean” chiral selection rules for the optical transitions between the twofold degenerate valence and conduction bands and the suppressed spin relaxation owing to the absence of a bands' spin splitting, resulting from the presence of a crystal inversion center. In time‐resolved circularly polarized luminescence for non‐resonant excitation in transverse magnetic field, we observe coherent spin precession of electrons and holes, providing direct access to their Landé factors and corroborating that the spin orientation of the charge carriers is preserved during the course of their energy relaxation. Importantly, for the same conditions we observe linear polarization of the emission which serves as unequivocal indication of electron and hole spin correlations in perovskites. Combined with the simple fabrication and the bright optical properties, these features make lead halide perovskites a prime material system for spintronic technologies.

## 4 Experimental Section

### Samples

The studied FA_0.9_Cs_0.1_PbI_2.8_Br_0.2_ bulk single crystal was grown out of solution with the inverse temperature crystallization technique.^[^ ^46^ ^]^ A solution of CsI, FAI, ${\mathrm{PbI}}_{2}$, and ${\mathrm{PbBr}}_{2}$ was mixed with GBL γ‐butyrolactone solvent. The solution was filtered and heated to $130^{\circ }C$, so the crystals were formed in the α‐phase.^[^ ^47^ ^]^ Single crystals were separated by filtration and drying. The α‐phase or black phase of FA_0.9_Cs_0.1_PbI_2.8_Br_0.2_ had a cubic crystal structure at room temperature. Further growth details are given in the Note S1 (Supporting Information). Since the crystal shows a *g*‐factor isotropy at low temperatures, its structure was also considered as cubic.^[^ ^17^ ^]^ The geometry with light wave vector k∥[001] was used in all optical experiments.

### Magneto‐Optical Measurements

For low‐temperature optical measurements a liquid helium cryostat with the temperature variable from 1.6 K up to 300 K was used. At T=1.6 K the sample was placed in superfluid helium, while at 4.2−30 K it was held in helium vapor. A superconducting magnet equipped with a pair of split coils could generate a magnetic field up to 5 T. The cryostat was rotated by $90^{\circ }$ to change the experimental geometry: The magnetic field parallel to k is denoted as $B_{F}$ (Faraday geometry) and perpendicular to k as $B_{V}$ (Voigt geometry).

### Photoluminescence and Photoluminescence Excitation Measurements

The time‐integrated photoluminescence spectrum (PL) was measured with a 0.5 m spectrometer equipped with a charge‐coupled‐devices (CCD) camera. For PLE, the PL intensity at the energy $E_{\det}=1.496$ eV was detected as a function of the excitation energy of a tunable titanium‐sapphire continuous wave laser. Scheme of the experimental setup is shown in Section S2 (Supporting Information).

### Time‐Resolved Photoluminescence

The spectrally resolved PL dynamics were measured using a spectrometer with 300 grooves mm^–1^ diffraction grating and a streak camera with 10 ps time resolution. Pulses with 200 fs duration and photon energies from 1.59  (780 nm) to 2.67 eV (465 nm) from a tunable Chameleon Discovery laser with a repetition rate of 80 MHz were used for PL excitation. The time‐integrated PL spectrum was obtained from a time integration of the PL dynamics. To study the effect of optical orientation, circularly ($\sigma^{+}/\sigma^{-}$) polarized excitation light was used and the circularly or linearly polarized emission was analyzed.

The dynamics of the full intensity (proportional to the population) showed a decay after pulse action with multiple recombination times ($\tau_{\mathrm{Ri}}$):

(7) $$I\left(t\right)=\sum_{i=1,2}I_{i}\left(0\right)\exp\left(-t/\tau_{\mathrm{Ri}}\right)$$

where $I_{i}\left(0\right)$ is the initial population of each component.

The dynamics of the optical orientation degree can be described by a decaying oscillatory function:

(8) $$P_{\mathrm{oo}}\left(t\right)=\sum_{i}P_{\mathrm{oo}}\left(0\right)\cos\left(\omega_{L,i}t\right)\exp\left(-t/\tau_{s,i}\right)$$

Here $P_{\mathrm{oo}}\left(0\right)$ is the spin polarization degree at zero time delay, the index i=e,h denotes the electron or hole component to the Larmor precession frequency $\omega_{L,i}$ and in the spin relaxation time $\tau_{s,i}$. The exciton Larmor precession in the degree of linear polarization is described by:

(9) $$P_{\mathrm{lin}}\left(t\right)=P_{\mathrm{lin}}\left(t=0\right)\cos\left(\omega_{L,X}t\right)\exp\left(-t/\tau_{X}\right)$$

### Theoretical Analysis

The band energies, matrix elements of velocity, and amplitudes of the optical transitions between $k_{0}$ and $k_{R}$ were calculated in the empirical tight‐binding approach. The tight‐binding approach with the parameters of ${\mathrm{CsPbI}}_{3}$ from ref. [42] was used. The matrix elements of velocity were calculated following ref. [48]. In calculations, a 50×50×50k‐mesh in 1/8 of the (cubic) Brillouin zone was taken. For more details see Section S8 (Supporting Information).

## Conflict of Interest

The authors declare no conflict of interest.

## Supporting information

Supporting Information

## Data Availability

The data that support the findings of this study are available from the corresponding author upon reasonable request.
