# Supplementary material for: Highly‐Polarized Emission Provided by Giant Optical Orientation of Exciton Spins in Lead Halide Perovskite Crystals
Source: Adv Sci (Weinh). 2024 Jun 17;11(31):2403691. doi: 10.1002/advs.202403691 (PMC11336922; doi:10.1002/advs.202403691)
Supplement: Supplementary file 1 — Supporting Information [file ADVS-11-2403691-s001.pdf]

ADVANCED SCIENCE
Open Access

Supporting Information

for *Adv. Sci.*, DOI 10.1002/advs.202403691

Highly-Polarized Emission Provided by Giant Optical Orientation of Exciton Spins in Lead Halide Perovskite Crystals

Nataliia E. Kopteva, Dmitri R. Yakovlev*, Eyüp Yalcin, Ilya A. Akimov, Mikhail O. Nestoklon, Mikhail M. Glazov, Mladen Kotur, Dennis Kudlacik, Evgeny A. Zhukov, Erik Kirstein, Oleh Hordiichuk, Dmitry N. Dirin, Maksym V. Kovalenko and Manfred Bayer*

Supplementary Information: Highly-polarized emission provided by giant optical orientation of exciton spins in lead halide perovskite crystals

Nataliia E. Kopteva, Dmitri R. Yakovlev, Eyüp Yalcin, Ilya A. Akimov, Mikhail O. Nestoklon, Mikhail M. Glazov, Mladen Kotur, Dennis Kudlacik, Evgeny A. Zhukov, Erik Kirstein, Oleh Hordiichuk, Dmitry N. Dirin, Maksym V. Kovalenko, and Manfred Bayer

S1. MATERIAL INFORMATION

The studied lead halide perovskite crystals belong to the $FAPbI_3$ material class. FA-based perovskites exhibit a low trap density (1.13×10^{10} cm^{-3}) and a low dark carrier density (3.9×10^{9} cm^{-3}) [S1, S2]. $FAPbI_3$ is chemically and thermally more stable compared to $MAPbI_3$ due to the possible decomposition of the latter to gaseous hydrogen iodide and methylammonium [S1]. However, pure $FAPbI_3$ suffers from structural instability originating from the large size of FA the cation which cannot be accommodated by the inorganic perovskite framework. This instability has been successfully resolved via partial, up to 15%, replacement of the large FA cation with the smaller caesium (Cs) together with the iodine (I) partial substitution by bromide (Br) [S3, S4]. As a result, the Goldschmidt tolerance factor [S5] t is tuned from 1.07 in $FAPbI_3$ closer to 1 in $FA_{0.9}Cs_{0.1}PbI_{2.8}Br_{0.2}$, where it is 0.98. The band gap of $FA_{0.9}Cs_{0.1}PbI_{2.8}Br_{0.2}$ at room temperature is 1.52 eV, slightly larger than the band gap of $FAPbI_3$ of 1.42 eV [S1, S6].

For crystal synthesis the inverse temperature crystallization technique is used [S1, S2]. For the growth, a solution of CsI, FAI (FA being formamidinium), PbI_2, and $PbBr_2$, with GBL γ-butyrolactone as solvent is mixed. This solution is then filtered and slowly heated to 130°C temperature, whereby single crystals are formed in the black phase of $FA_{0.9}Cs_{0.1}PbI_{2.8}Br_{0.2}$, following the reaction

$$\mathrm{PbI_2 + PbBr_2 + FAI + CsI} \overset{[GBL]}{\rightarrow} \mathrm{FA_{0.9}Cs_{0.1}PbI_{2.8}Br_{0.2} + R}.$$

Afterwards, the crystals are separated by filtering and drying. A typical crystal as used for this study has a size of about 2 mm. The crystallographic analysis suggests that one of the principal axes a, b, c is normal to the front facet, thus pointing along the optical axis. In cubic approximation $a = b = c$. The pseudo-cubic lattice constant for hybrid organic perovskites (HOP) is around 0.63 nm [S7], but was not determined for this specific sample.

S2. EXPERIMENTAL DETAILS

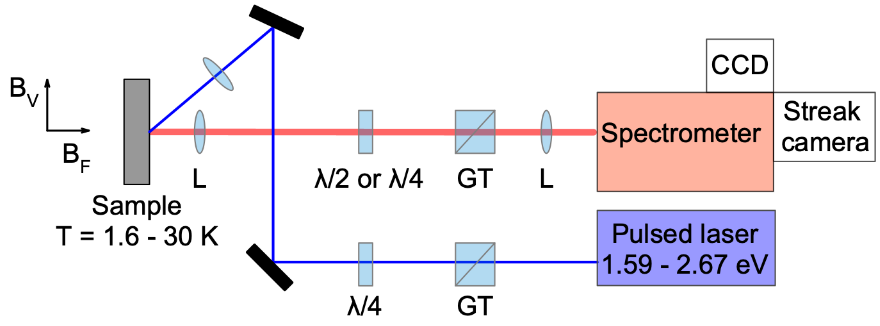

FIG. S1. Schema of the experimental setup for photoluminescence measurements. L is the lens, GT is the Glan-Tompson polarizer, $\lambda/2$ and $\lambda/4$ are the linear and circular polarizers.

For low-temperature optical measurements we use a liquid helium cryostat with the temperature variable from 1.6 K up to 300 K. At $T = 1.6$ K the sample is placed in superfluid helium, while at $4.2 - 30$ K it is held in helium vapor.

A superconducting magnet equipped with a pair of split coils can generate magnetic fields up to 5 T. The cryostat is rotated by 90° to change the experimental geometry: The magnetic field parallel to $\mathbf{k}$ is denoted as B_F (Faraday geometry) and perpendicular to $\mathbf{k}$ as B_V (Voigt geometry) as shown in Fig. S1.

The time-integrated photoluminescence (PL) spectrum was measured with a 0.5 m spectrometer equipped with a charge-coupled-device (CCD) camera. For PL excitation measurements, the PL intensity at the energy $E_{det} = 1.496$ eV was detected as a function of the excitation energy of a tunable titanium-sapphire continuous wave laser.

The spectrally resolved PL dynamics were measured using a spectrometer with a 300 grooves/mm diffraction grating and a streak camera with 10 ps time resolution. Pulses with 200 fs duration and photon energies from 1.59 eV (780 nm) to 2.67 eV (465 nm) from a tunable Chameleon Discovery laser with the repetition rate of 80 MHz were used for PL excitation. The time-integrated PL spectrum is obtained from time integration of the PL dynamics. To study the effect of optical orientation, we used circularly (σ^+/σ^-) polarized excitation light and analyzed the circularly or linearly polarized emission (see Fig. S1).

S3. TIME-RESOLVED PHOTOLUMINESCENCE IN THE MICROSECOND RANGE

The time-resolved photoluminescence (TRPL) detection technique with high temporal resolution of 10 ps by means of a streak-camera covers a time range limited to 2 ns. In order to measure longer times, we use pump-probe differential reflectivity or TRPL using a time-of-flight computer board. In $FA_{0.9}Cs_{0.1}PbI_{2.8}Br_{0.2}$ at $T = 6$ K the decay of the population dynamics measured by pump-probe differential reflectivity occurs with a time constant of 450 ps [S8]. This is in good agreement with the $\tau_{R2} = 380 - 840$ ps obtained from streak camera measurements, see Figure 1c. However, the PL dynamics contain also long decay times, which are not related to exciton recombination.

The long-lived recombination dynamics up to 100 μs are measured by the TRPL technique. Here, the PL is excited by a pulsed laser with parameters: photon energy of 2.33 eV (wavelength of 532 nm), pulse duration of 5 ns, repetition rate of 10 kHz, and average excitation power of 8 μW. The detection energy was selected by a Jobin-Yvon U1000 double monochromator (1 meter focal length). The signal was detected using an avalanche photodiode connected to time-of-flight card with time resolution of 30 ns.

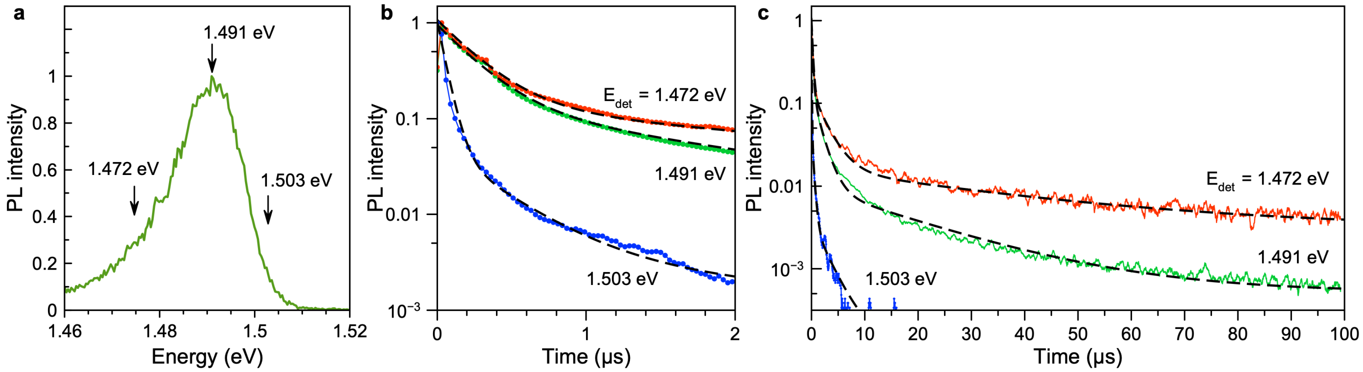

FIG. S2. (a) Time-integrated PL measured for pulsed excitation with $E_{exc} = 2.33$ eV and repetition rate of 10 kHz. $T = 1.6$ K. Time-resolved PL measured at various energies in 2 μs range (b) and in 100 μs range (c). PL dynamics fitted with a multi-exponential function with decay times given in Table S1. The fits are shown by black dashed lines.

TABLE S1. Recombination times for $FA_{0.9}Cs_{0.1}PbI_{2.8}Br_{0.2}$, measured at different spectral positions for $T = 1.6$ K.

E_{det} (eV)	τ_1 (ns)	τ_2 (ns)	τ_3 (μs)	τ_4 (μs)
1.503	55	370	3.0	-
1.491	-	250	1.6	20
1.472	-	200	3.3	44

Figure S2 shows the recombination dynamics in a $FA_{0.9}Cs_{0.1}PbI_{2.8}Br_{0.2}$ crystal, detected at various spectral energies. The time-integrated PL spectrum measured for pulsed laser excitation is shown in Figure S2a. The arrows

indicate the detection energy of the photoluminescence dynamics. The experiments were performed at the temperature of $T = 1.6$ K. The recombination dynamics cover a broad temporal range up to 100 μs. They cannot be fitted by a monoexponential decay evidencing that several recombination processes are involved. The PL dynamics comprise three exponential decays in addition to the short exciton recombination decay, which is too fast to be resolved in this experiment, see Figures S2b,c. The long recombination dynamics can be associated with the following processes: (i) Recombination of electrons and holes localized at different crystal sites with a significant dispersion in their separation lengths [S8–S12], (ii) Carrier trapping and detrapping processes [S13], (iii) Polaron formation [S14], (iv) Slow carrier diffusion from the sample surface to the crystal depth [S15]. We suggest that in the crystal studied in our paper the first two mechanisms are most probable, while the clarification of the role of the specific mechanisms requires comprehensive studies, which go beyond the scope of the present study. All these mechanisms suggest that the long living electrons and holes are present in perovskite crystals, whose lifetimes greatly extend the recombination dynamics of excitons.

S4. SPECTRAL DEPENDENCE OF OPTICAL SPIN ORIENTATION

$P_{\rm oo}$ has strong dependence on the detection energy, as shown in Fig. S3. The maximal $P_{\rm oo}$ is observed on the exciton resonance at 1.506 eV. It decreases for localized carriers (see Fig. S3) forming the low energy spectral line. To detect $P_{\rm oo}$ of processes with recombination time longer than the time repetition rate of fs laser (of about 13 ns), we employ the continuous-wave excitation technique, results are in Ref. [S16].

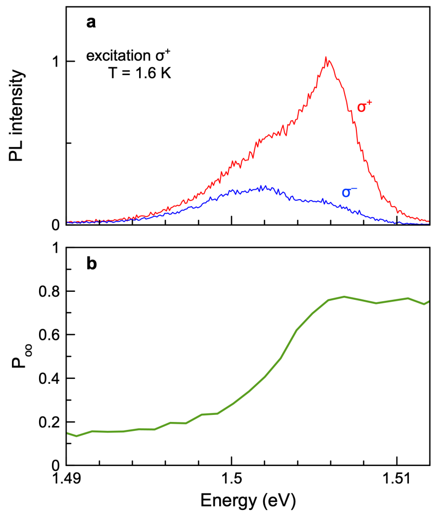

FIG. S3. Spectral dependence of $P_{\rm oo}$ at $T = 1.6$ K. (a) Red and blue lines are the photoluminescence spectra integrated over the exciton lifetime in σ^+ and σ^- polarization, respectively, for σ^+ polarized excitation. $E_{\rm exc} = 1.669$ eV and $P = 10$ mW/cm^2. (b) Spectral dependence of the optical orientation degree.

S5. OPTICAL ORIENTATION OF EXCITONS MEASURED FOR RESONANT EXCITATION

In order to study the direct optical orientation of excitons, we use resonant excitation in pump-probe differential reflectivity. We use a laser system generating pulses with 1.5 ps duration and repetition rate of 75 MHz. The pump and probe photon energies were the same and were set to the exciton resonance at 1.503 eV. The pump was σ^+ circularly polarized, its intensity was modulated by an electro-optical modulator at a frequency of 100 kHz. The probe beam was either σ^+ or σ^- circularly polarized. It was reflected from the sample and sent on a photodiode, which output was analyzed by a lock-in amplifier. The temporal resolution was about 2 ps. Note that this technique has a higher time resolution than TRPL. Also both the population dynamics and spin dynamics of excitons and photogenerated carriers can be measured.

Figure S4a shows the dynamics of the differential reflectivity ($\Delta R/R$) signal detected in σ^+ polarization (red line, denoted as I^{++}) and σ^- polarization (blue line, I^{+-}) polarization of the probe. I^{++} decays in time, but I^{+-} rises.

From a double-exponential fit of the full intensity ($I^{++} + I^{+-}$) shown in Figure S4b, we obtain a fast decay time of 150 ps and a slow decay time of 4 ns. The fast time can be assigned to the exciton lifetime, and the slow time to the recombination of spatially separated resident electrons and holes. the dynamics of the optical orientation degree, $P_{\rm oo}(t)$, are shown in Figure S4c. Its maximal value at a time delay of a few picoseconds reaches 0.95 (i.e. 95%), which in fact is close to the maximal possible value of 100%. Note, that we do not show or use in our analysis the strong signal during the time overlap of the pump and probe pulses.

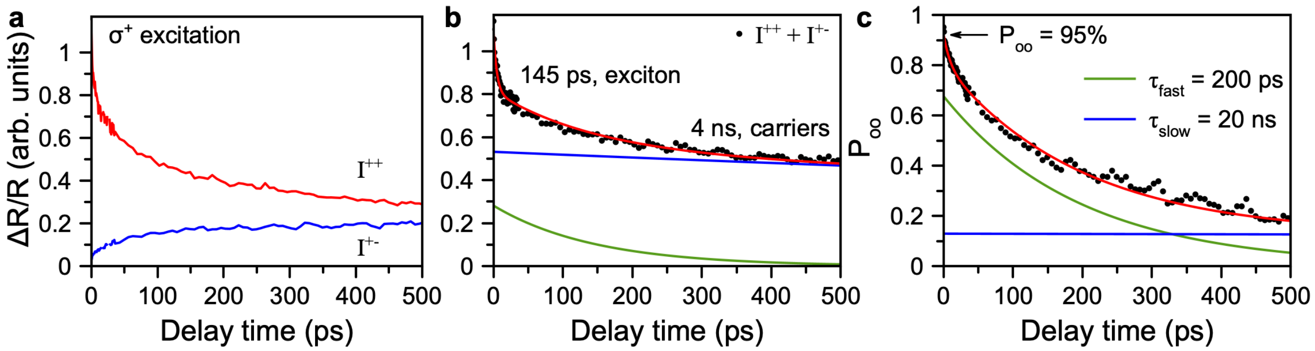

FIG. S4. Spin dynamics measured by polarized pump-probe reflectivity at $T = 1.6$ K. (a) Dynamics of differential reflectivity ($\Delta R/R$) measured for σ^+ polarized pump, with the detection probe pulses either σ^+ (red line) or σ^- (blue line) polarized. The laser photon energy $E_{\rm exc} = E_{\rm det} = 1.503$ eV matches the exciton resonance. The pump power is 15 mW and the probe power is 0.6 mW. (b) The calculated full intensity ($I^{++} + I^{+-}$) shown by the black dots with the recombination time of the exciton $\tau_{\rm X} = 150$ ps (green) and of spatially separated carriers $\tau_{\rm R,2} = 4$ ns (blue). The red line is the sum fit including the exciton and resident carriers contributions. (c) Dynamics of the optical orientation $P_{\rm oo}(t)$ (dots) calculated from the data from panel (a) with Eq. (1). the red line is a bi-exponential fit. Green and blue lines show the fast and slow components of the $P_{\rm oo}(t)$ dynamics.

S6. EFFECT OF EXCITATION DENSITY

Here we present experimental results on the modification of the recombination and spin dynamics of excitons and resident carriers with increasing excitation density. It allows us to better understand the interplay of the exciton and carrier contributions to the emission at the same spectral energy of 1.506 eV. We show below that in this case the dynamics of the optical orientation degree is determined not by the spin relaxations time, as it is common when only one recombination process is involved, but rather by the fast recombination of excitons. Therefore one has to be careful with interpretations. Note that this situation is rather unusual for the spin physics of semiconductors, and we are not aware of a corresponding discussion in literature.

In the main text, we present experimental data measured at small excitation densities of 10 to 30 mW/cm^2. The excitation density of 10 mW/cm^2 corresponds to the exciton density of 10^{13} cm^{-3}. The density of photogenerated excitons is low, so that the exciton-exciton interaction can be neglected. Note, that for the large excitation densities, especially for the nanostructures, the exciton-exciton interaction may be strong [S17, S18]. Here we show results for recombination and $P_{\rm oo}(t)$ dynamics, measured at excitation densities varied across a wide range from 10 up to 600 mW/cm^2. Figure S5a compares the recombination dynamics at 10 mW/cm^2 (black line) and 600 mW/cm^2 (blue line). One can see that with increasing the excitation density the initial decay becomes slower, the decay time increases from 55 ps up to 200 ps. By contrast, the slow contribution to the dynamics becomes faster, the corresponding decay time shortens from 840 ps down to 200 ps. A redistribution of intensity between the fast and slow contributions also takes place. As a result, the initial decay of PL intensity by about 95% becomes monoexponential at $P = 600$ mW/cm^2 with a decay time of 200 ps. The dependences of the recombination times $\tau_{\rm R1}$ and $\tau_{\rm R2}$ on P are shown in Figure S5c. We assign the longer recombination time $\tau_{\rm R2}$ to the recombination of spatially separated electrons and holes. As their concentrations increase at higher excitation densities, the separation between them reduces so that their recombination becomes faster. We suggest that the increase of $\tau_{\rm R1}$ time with growing P evidences an increasing contribution of separated electrons and holes, compared to the exciton recombination. At the largest excitation density the PL dynamics is mostly related to electron-hole recombination.

Figure S5b shows the dynamics of optical orientation ($P_{\rm oo}(t)$) measured at $P = 10$ mW/cm^2 and 600 mW/cm^2. The dependence at $P = 10$ mW/cm^2 (blue dots) is the same as shown in the main text in Figure 2b, but here it

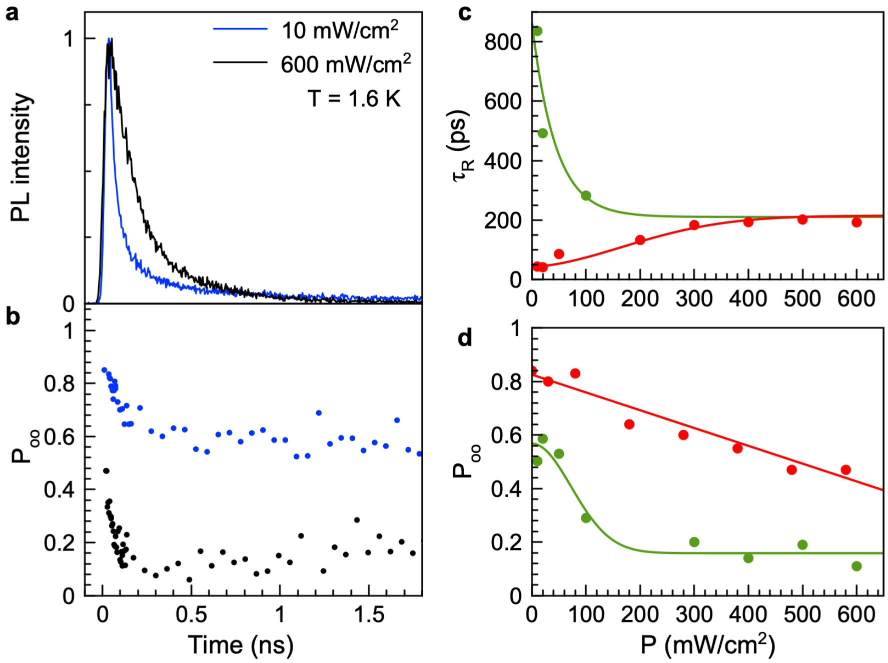

FIG. S5. (a) TRPL signals for $P = 10\,\mathrm{mW/cm^2}$ (blue line) and for $P = 600\,\mathrm{mW/cm^2}$ (black line). $T = 1.6K$, $E_{\mathrm{det}} = 1.506$ eV, $E_{\mathrm{exc}} = 1.675$ eV. (b) Time-resolved P_{oo} for $P = 10\,\mathrm{mW/cm^2}$ (blue dots) and for $P = 600\,\mathrm{mW/cm^2}$ (black dots). (c) τ_{R1} (red dots) and τ_{R2} (green dots) dependences on the excitation density. Lines are guides to the eye. (d) Optical orientation of excitons (measured at $t = 0$ ns, red dots) and of localized carriers (measured at $t = 1.5$ ns, green dots) as function of excitation density. Lines are guides to the eye.

extends over a longer temporal range up to 1.8 ns, highlighting the almost negligible decay of polarization across the whole time range. One can see, that at the initial time moment right after excitation $P_{\mathrm{oo}}(t = 0) = 0.85$ and then decreases and saturates at a level of about 0.60. The transition lasts about 55 ps, i.e. the same time that we have measured for the exciton lifetime in the recombination dynamics $\tau_{\mathrm{X}} = 55$ ps. Therefore, the dynamics of $P_{\mathrm{oo}}(t)$ reflect the fact that the excitons with the very high optical orientation degree of 0.85 recombine and at longer time the orientational degree is given by the smaller values typical for resident carriers. Therefore, we observe here a rather unusual situation where the dynamics of $P_{\mathrm{oo}}(t)$ is determined not by the spin relaxation time, but by the recombination time.

At the maximal $P = 600$ mW/cm^2 used in experiment, the initial optical orientation decreases to $P_{\mathrm{oo}}(t = 0) = 0.50$. It drops over a time range of 150 ps after which it reaches the saturation level of about 0.15, see the black dots in Figure S5b. The power dependences of $P_{\mathrm{oo}}(t = 0)$ and $P_{\mathrm{oo}}(t = 1.5$ ns) are shown in Figure S5d. One can see that the initial degree decreases steadily with increasing power density from 0.85 down to 0. while the degree at longer delays, which is contributed by resident carriers, shows the strongest decrease from 0.60 down to 0.20 up to powers of 100 mW/cm^2. The decrease of the saturation level is related to an increase of the (short) exciton recombination time τ_{R1} and is in line with Eq. (2) of the main text. Indeed, at low pumping, the factor $\nu = \tau_s/(\tau_{\mathrm{X}} + \tau_s) \approx 0.8$ (from the experimental data), in consistency with $\tau_{\mathrm{X}} \equiv \tau_{\mathrm{R1}} = 55$ ps and $\tau_s = 220$ ps as discussed in the main text. It can describe the reduction of P_{oo} from 0.85 to 0.60, while the factor $\nu \approx 0.5$ corresponds to $\tau_{\mathrm{X}} \equiv \tau_{\mathrm{R1}} = 200$ ps at high pump powers. Additional mechanisms such as a reduction of the exciton spin relaxation time at elevated densities cannot be excluded as well.

In our experiments the sample is in direct contact with superfluid liquid helium. Therefore, we expect that in the whole range of used power heating effects of the crystal lattice (phonon system) are very small. But exciton and carrier temperatures can indeed be elevated. If the origin of the smaller P_{oo} at higher excitation density is a local heating by the laser, then $P_{\mathrm{oo}}(t = 0) = 0.5$ at 600 mW/cm^2 corresponds to 10 K on temperature dependence (see Figure 2e in the main text). The exciton recombination time at 10 K does not increase in comparison to 1.6 K and remains about 60 ps. However, at 1.6 K and 600 mW/cm^2 pump fluence the recombination time is 200 ps. From that we also conclude that the increase of the pump fluence does not contribute significantly to the sample heating. It is also consistent with the absence of wave vector dependent Rashba and Dresselhaus terms, otherwise, one can naturally expect an enhancement of the spin relaxation with increasing the temperature.

S7. THEORY OF EXCITON AND CARRIER SPIN PRECESSION IN MAGNETIC FIELD

The cubic perovskites represent a special class of systems where the bottom conduction and topmost valence bands are ‘simple’, two-fold spin degenerate. This results in quite specific exciton spin dynamics in magnetic field, controlled by the interplay of the exchange interaction and the Zeeman splittings of individual charge carriers. The basics of exciton spin physics in such a band structure in the presence of a transverse magnetic field were described in [S19], see also [S20]. Here we present the theory with emphasis on the temporal dynamics of the exciton spin polarization. We would like to point out that the spin properties of bulk perovskite with cubic symmetry are quite different as compared with conventional III-V or II-VI semiconductors. The simple band structure of perovskites results in entirely new spin properties of the excitons. In bulk III-V and II-VI semiconductors, the spin dynamics of excitons are difficult to study because the complex structure of the valence band and polariton effects play an important role [S19]. In quantum wells based on conventional semiconductors, the exchange interaction separates the bright exciton states (±1) from the dark exciton states (±2) and strongly suppresses the in-plane magnetic field effect. In contrast to conventional III-V and II-VI semiconductors, in halide perovskites the bands are “simple” and the magnetic field efficiently couples the states of the bright exciton triplet [S21, S22].

A. Temporal dynamics

We recall that both the top valence and the bottom conduction bands in the lead halide perovskite semiconductors are two fold spin degenerate and can be conveniently described by spin-1/2 operators. The Hamiltonian of an electron-hole pair in perovskites with cubic symmetry in an external magnetic field $\mathbf{B}$ can be written as:

$$\hat{H} = \mu_{\rm B} g_{\rm e} \mathbf{s}_{\rm e} \mathbf{B} + \mu_{\rm B} g_{\rm h} \mathbf{s}_{\rm h} \mathbf{B} + \Delta_{\rm X} \hat{\mathbf{s}}_{\rm e} \cdot \hat{\mathbf{s}}_{\rm h}. \tag{S1}$$

Here $\mu_{\rm B}$ is the Bohr magneton, $\mathbf{B}$ is the external magnetic field, $\Delta_{\rm X}$ is the electron-hole exchange splitting, $\mathbf{s}_{\rm e}$ and $\mathbf{s}_{\rm h}$ are the electron and hole spin-1/2 operators. Note that the exchange interaction, in agreement with the cubic symmetry, can be recast as

$$\hat{\mathbf{s}}_{\rm e} \cdot \hat{\mathbf{s}}_{\rm h} = \frac{1}{2} \hat{\mathbf{J}}^2 - \frac{3}{4},$$

where $\hat{\mathbf{J}} = \hat{\mathbf{s}}_{\rm e} + \hat{\mathbf{s}}_{\rm h}$ is the total spin operator of the electron hole pair and $\hat{\mathbf{J}}^2 = J(J+1)$. The J takes one of the two values 0 or 1. In what follows, we choose the eigenstates of the exchange interaction Hamiltonian in the form of $|J, J_z\rangle$ as:

$$\phi_1 = |1, +1\rangle = |\uparrow, \Uparrow\rangle, \tag{S2a}$$

$$\phi_2 = |1, 0\rangle = \frac{1}{\sqrt{2}} (|\uparrow, \Downarrow\rangle + |\downarrow, \Uparrow\rangle), \tag{S2b}$$

$$\phi_3 = |1, -1\rangle = |\downarrow, \Downarrow\rangle, \tag{S2c}$$

$$\phi_4 = |0, 0\rangle = \frac{1}{\sqrt{2}} (|\uparrow, \Downarrow\rangle - |\downarrow, \Uparrow\rangle). \tag{S2d}$$

Here, the up and down arrows indicate the spin: $+1/2$ and $-1/2$, respectively. The symbols $\uparrow$ and $\Uparrow$ correspond to electron and hole spins, respectively. In what follows, it is convenient to take the z-axis along the $\mathbf{k}$-vector of light. At $B = 0$ the eigenstates are the spin singlet, $J = 0$, with the energy $-3\Delta_{\rm X}/4$ and the spin triplet, $J = 1$, with the energy $\Delta_{\rm X}/4$, the splitting between these states is $\Delta_{\rm X}$. The states with $J_z = \pm 1$ (ϕ_1, ϕ_3) are optically active in $\sigma^\pm$ circular polarizations. The exciton state $|1, 0\rangle$ has the dipole moment along the z-axis (“longitudinal” exciton) and dark in this geometry. The state $|0, 0\rangle$ is dark (spin forbidden).

In the presence of a magnetic field, the states with the same component of the total spin along the magnetic field are mixed, and the states with different components of the total spin get split. Particularly, in the Voigt geometry where $\mathbf{B} = (B_{\rm V}, 0, 0) \parallel x$, the Hamiltonian (S1) takes the form

$$\hat{H} = \frac{1}{2\sqrt{2}} \begin{pmatrix} 2\sqrt{2}\Delta_{\rm X} & \mu_{\rm B} g_{\rm V,X} B_{\rm V} & 0 & -\mu_{\rm B} g_{\rm V,DX} B_{\rm V} \\ \mu_{\rm B} g_{\rm V,X} B_{\rm V} & 2\sqrt{2}\Delta_{\rm X} & \mu_{\rm B} g_{\rm V,X} B_{\rm V} & 0 \\ 0 & \mu_{\rm B} g_{\rm V,X} B_{\rm V} & 2\sqrt{2}\Delta_{\rm X} & \mu_{\rm B} g_{\rm V,DX} B_{\rm V} \\ -\mu_{\rm B} g_{\rm V,DX} B_{\rm V} & 0 & \mu_{\rm B} g_{\rm V,DX} B_{\rm V} & 0 \end{pmatrix}, \tag{S3}$$

where $g_{\mathrm{V,X}} = g_{\mathrm{V,e}} + g_{\mathrm{V,h}}$ is the so-called bright exciton g-factor and $g_{\mathrm{V,DX}} = g_{\mathrm{V,e}} - g_{\mathrm{V,h}}$ is the dark exciton g-factor. Note that while in the Voigt geometry all excitonic states are optically active, in general, see the eigenfunctions of the Hamiltonian below, we use the notations of the bright and dark exciton g-factors in analogy with the Faraday geometry. The Larmor frequency of the bright exciton is defined by $\omega_{\mathrm{L,X}} = \mu_{\mathrm{B}} g_{\mathrm{V,X}} B_{\mathrm{V}}/\hbar$.

The energies of the exciton states in the presence of the field read

$$E_{\mathrm{I}} = \frac{1}{2}\left(\Delta_{\mathrm{X}} - \sqrt{\Delta_{\mathrm{X}}^2 + (\mu_{\mathrm{B}} g_{\mathrm{V,DX}} B_{\mathrm{V}})^2}\right), \tag{S4a}$$

$$E_{\mathrm{II}} = \frac{1}{2}\left(\Delta_{\mathrm{X}} + \sqrt{\Delta_{\mathrm{X}}^2 + (\mu_{\mathrm{B}} g_{\mathrm{V,DX}} B_{\mathrm{V}})^2}\right), \tag{S4b}$$

$$E_{\mathrm{III}} = \Delta_{\mathrm{X}} - \frac{1}{2}\mu_{\mathrm{B}} g_{\mathrm{V,X}} B_{\mathrm{V}}, \tag{S4c}$$

$$E_{\mathrm{IV}} = \Delta_{\mathrm{X}} + \frac{1}{2}\mu_{\mathrm{B}} g_{\mathrm{V,X}} B_{\mathrm{V}}. \tag{S4d}$$

The eigenfunctions can be conveniently expressed as superpositions of the basic states (S2) as

$$|\mathrm{i}\rangle = \sum_j a_{\mathrm{i},j}\phi_j, \tag{S5}$$

where the Roman index i = I, II, III or IV denotes the eigenstates in the magnetic field, and the arabic subscript $j = 1, 2, 3,$ and 4 denotes the basic functions in (S2). The decomposition coefficients $a_{\mathrm{i},j}$ can be recast in column-vector form:

$$a_{\mathrm{I},j} = \begin{pmatrix} \frac{1}{2}\sqrt{1-\Delta_{\mathrm{X}}/C} \\ 0 \\ -\frac{1}{2}\sqrt{1-\Delta_{\mathrm{X}}/C} \\ \frac{\mu_{\mathrm{B}} g_{\mathrm{V,DX}} B_{\mathrm{V}}}{\sqrt{2C(C-\Delta_{\mathrm{X}})}} \end{pmatrix}, \quad a_{\mathrm{II},j} = \begin{pmatrix} -\frac{1}{2}\sqrt{1+\Delta_{\mathrm{X}}/C} \\ 0 \\ \frac{1}{2}\sqrt{1+\Delta_{\mathrm{X}}/C} \\ \frac{\mu_{\mathrm{B}} g_{\mathrm{V,DX}} B_{\mathrm{V}}}{\sqrt{2C(C+\Delta_{\mathrm{X}})}} \end{pmatrix}, \quad a_{\mathrm{III},j} = \begin{pmatrix} \frac{1}{2} \\ -\frac{\sqrt{2}}{2} \\ \frac{1}{2} \\ 0 \end{pmatrix}, \quad a_{\mathrm{IV},j} = \begin{pmatrix} \frac{1}{2} \\ \frac{\sqrt{2}}{2} \\ \frac{1}{2} \\ 0 \end{pmatrix}, \tag{S6}$$

with $C = \sqrt{\Delta_{\mathrm{X}}^2 + (\mu_{\mathrm{B}} g_{\mathrm{V,DX}} B_{\mathrm{V}})^2}$.

The dependences of the exciton level energies on B_{V} are shown in Figure S6a for the case of zero exchange interaction $\Delta_{\mathrm{X}} = 0$ meV. In that case, a linear-in-field Zeeman splitting is seen for all four states. In the presence of a significant exchange splitting, $\Delta_{\mathrm{X}} = 0.42$ meV, the pair of states III and IV show a linear-in-B_{V} Zeeman splitting. The pair of states I and II demonstrate a linear-in-field splitting only at high fields with an offset given by Δ_{X} for $B_{\mathrm{V}} \to 0$, as shown in Figure S6d.

In order to calculate the time- and polarization-resolved intensity of the photoluminescence following polarized excitation by a short laser pulse, we resort to a coherent model and disregard both the finite lifetime of excitons and the spin relaxation processes. Accordingly, we write the wavefunction of the system $\Psi(t)$ as a superposition of the eigenstates $|\mathrm{i}\rangle$ in Eq. (S5):

$$\Psi(t) = \sum_{\mathrm{i=I}}^{\mathrm{IV}} C_{\mathrm{i}} |\mathrm{i}\rangle \exp(-i\omega_{\mathrm{i}} t), \quad \omega_{\mathrm{i}} = E_{\mathrm{i}}/\hbar. \tag{S7}$$

The decomposition coefficients C_{i} are determined by the initial conditions: excitation of the system by a circularly polarized light renders excitons in the ϕ_1 or ϕ_3 states, depending on the photon helicity, and the initial state $(\phi_1 - \phi_3)/\sqrt{2}$ corresponds to horizontal polarization along B_{V}, marked by $\|$. In the same fashion, $(\phi_1 + \phi_3)/\sqrt{2}$ describes the vertical, marked by $\perp$, polarized excitation. Similarly, the intensity of the emission in the given polarization is determined by the absolute value squared of the $\Psi(t)$ projection onto the correspondingly polarized state. In our experiments, the intensities in the σ^+, σ^-, $\|$ and $\perp$ polarizations were measured after σ^+ polarized excitation. They

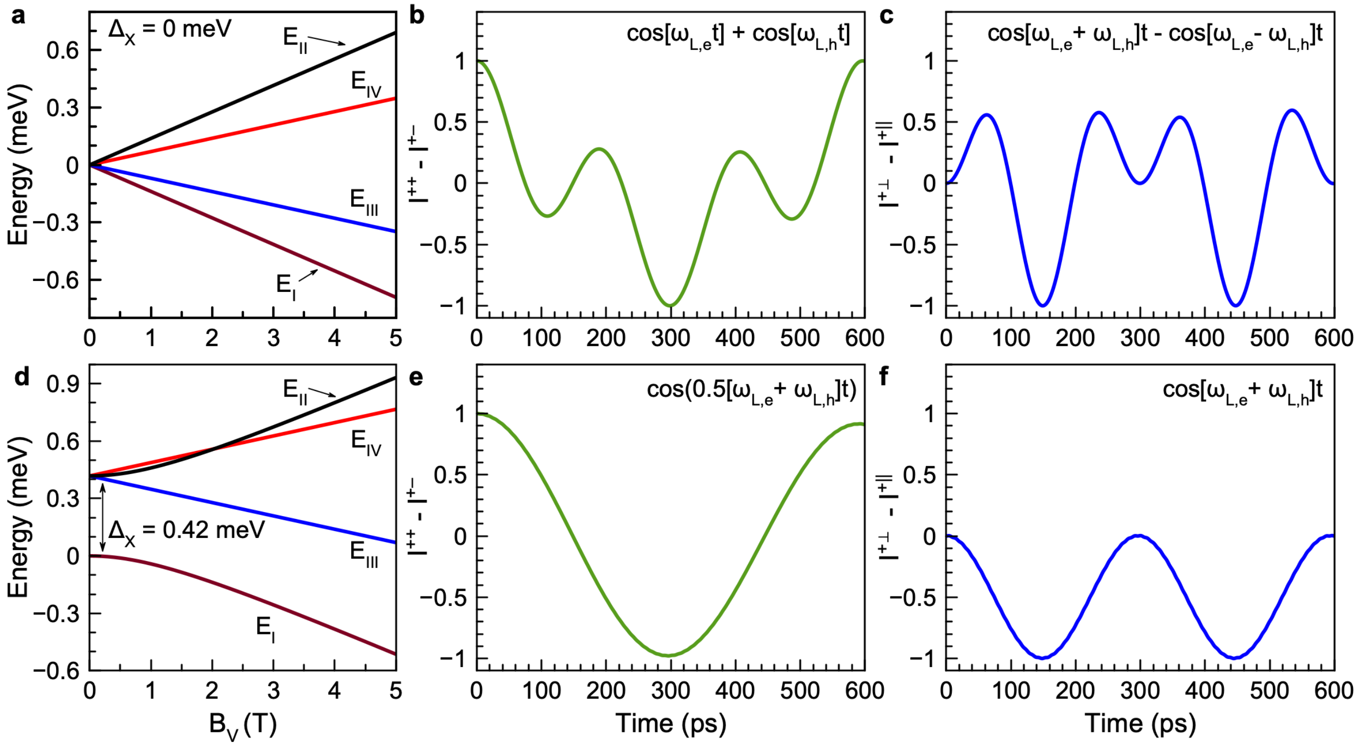

FIG. S6. Panels (a) and (d) show the energy levels of the excitons as a function of the magnetic field B_V, calculated for $\Delta_\mathrm{X} = 0\,\mathrm{meV}$ (a) and $\Delta_\mathrm{X} = 0.42\,\mathrm{meV}$ (d). Panels (b) and (e) present the dynamics of the signal $I^+ - I^-$ calculated for $\Delta_\mathrm{X} = 0\,\mathrm{meV}$ (b) and $\Delta_\mathrm{X} = 0.42\,\mathrm{meV}$ (e). Panels (c) and (f) show the dynamics of $I^\mathrm{V} - I^\mathrm{H}$ calculated for $\Delta_\mathrm{X} = 0\,\mathrm{meV}$ (c) and $\Delta_\mathrm{X} = 0.42\,\mathrm{meV}$ (f). Calculations are done for $g_{\mathrm{e,V}} = +3.48$, $g_{\mathrm{h,V}} = -1.15$, $B_\mathrm{V} = 0.1$ T.

read

$$I^{++} = \left|\sum_\mathrm{i} |a_{\mathrm{i},1}|^2 e^{-i\omega_\mathrm{i} t}\right|^2, \tag{S8a}$$

$$I^{+-} = \left|\sum_\mathrm{i} a_{\mathrm{i},1} a_{\mathrm{i},3} e^{-i\omega_\mathrm{i} t}\right|^2, \tag{S8b}$$

$$I^{+\parallel} = \left|\sum_\mathrm{i} (a_{\mathrm{i},1}^2 + a_{\mathrm{i},1} a_{\mathrm{i},3}) e^{-i\omega_\mathrm{i} t}\right|^2, \tag{S8c}$$

$$I^{+\perp} = \left|\sum_\mathrm{i} (a_{\mathrm{i},1}^2 - a_{\mathrm{i},1} a_{\mathrm{i},3}) e^{-i\omega_\mathrm{i} t}\right|^2. \tag{S8d}$$

Overall, the calculated intensities in Figure S6 show, in general, a complex oscillatory dependence on time related to the superposition of quantum beats with the frequencies following from Eqs. (S4). For the beats in circular polarization, simplified analytical expressions can be derived in the limit of negligible exchange, $\Delta_\mathrm{X} \to 0$, where

$$I^{++} - I^{+-} \propto \cos(\omega_{\mathrm{L,e}} t) + \cos(\omega_{\mathrm{L,h}} t), \tag{S9}$$

here $\omega_{\mathrm{L,e(h)}} = g_{\mathrm{e(h)}} \mu_\mathrm{B} B_\mathrm{V}/\hbar$ are the electron and hole Larmor precession frequencies. Naturally, in the case of $\Delta_\mathrm{X} \to 0$, excitonic effects are absent and the spin precessions observed correspond to those of the electron and hole spins with corresponding Larmor frequencies. in agreement with the numerical result in Figure S6b. Such situation also holds for the "long-living" TRPL signal caused by unbound electrons and holes, see Figure 3a of the main text. If the exchange splitting is large as compared to the Zeeman splitting, see $\Delta_\mathrm{X} > \hbar\omega_{\mathrm{L,X}}$, then we can disregard the admixture of the

singlet exciton and analyze the spin beats of the triplet of states described by the Zeeman Hamiltonian

$$\mathcal{H}_3 = \mu_B \frac{g_{\mathrm{V,X}}}{2} B_{\mathrm{V}} \hat{L}_x, \tag{S10}$$

where $\hat{L}_x$ is the matrix of the x-component of the angular momentum 1. Thus, the exciton pseudospin precesses with the frequency $\omega_{\mathrm{L,X}}/2 = \mu_B g_{\mathrm{V,X}} B_{\mathrm{V}}/(2\hbar)$ and

$$I^{++} - I^{+-} \propto \cos(\omega_{\mathrm{L,X}} t/2), \tag{S11}$$

in agreement with the beats calculated numerically in Figure S6e for weak magnetic fields. In case of a strong exchange splitting the $I^{++} - I^{+-}$ signal oscillates with half the Larmor frequency of the Zeeman splitting of the bright exciton $\omega_{\mathrm{L,X}}/2$.

Similarly, in the limit of weak exchange interaction the beats in linear polarization can be described by the simple expression

$$I^{+\perp} \propto 1 + \cos[(\omega_{\mathrm{L,e}} + \omega_{\mathrm{L,h}})t], \quad I^{+\parallel} \propto 1 + \cos[(\omega_{\mathrm{L,e}} - \omega_{\mathrm{L,h}})t]. \tag{S12}$$

The vertically linearly polarized component of the PL oscillates with the Larmor frequency of the bright exciton and the horizontally linearly polarized component with the dark exciton Larmor frequency. So, the signal $I^{\perp} - I^{\parallel}$ contains two oscillating contributions at the $\omega_{\mathrm{L,e}} + \omega_{\mathrm{L,h}}$ and $\omega_{\mathrm{L,e}} - \omega_{\mathrm{L,h}}$ frequencies, as shown in the Figure S6c. In the case of strong exchange interaction the analysis within the Hamiltonian (S10) gives rise to the beats

$$I^{+\perp} \propto 0.5(1 + \cos[\omega_{\mathrm{L,X}} t]), \quad I^{+\parallel} \propto 1, \tag{S13}$$

in agreement with the numerical calculations. In the experiments, exciton beats are seen in the linear polarization, see Figure 4c, and are reasonably well described by Eq. (S13).

We stress that the expressions for the temporal dependence of the intensities are derived within a fully coherent approach that disregards the spin relaxation and population relaxation of excitons. Phenomenologically, these can be included by the exponential factors $\exp(-t/\tau_{\mathrm{X}})$ to account for the exciton recombination and $\exp(-t/\tau_s)$ to account for the spin relaxation.

The model described above can be applied to the analysis of both the fast dynamics of excitons with large exchange splitting and charge carriers with negligible exchange interaction. The exciton dynamics occur mainly on the exciton radiative lifetime scale under the influence of the exchange interaction and Zeeman splittings. The dynamics of long-living electrons and holes is defined by the Zeeman splittings of individual charge carriers.

B. Electron and hole spin correlations in the linear polarization of emission

It is instructive to relate the linear polarization of emission with the correlators of electron and hole spins. To that end, we focus on the triplet $J = 1$ which consists, as described above, of two states active in $\sigma^{\pm}$ polarization for light propagating along the z-axis and the "longitudinal" state. This triplet is described by the operators $\hat{L}_\alpha$ of the angular momentum 1, where $\alpha = x, y, z$. One can directly check that the degree of *circular* polarization of single exciton emission is given by the quantum mechanical average

$$P_{\mathrm{oo}} = \langle \hat{L}_z \rangle, \tag{S14a}$$

while the degrees of *linear polarization* in the sets of axes (xy) and $(x'y')$ rotated by 45° relative to each other are given by

$$P_{\mathrm{lin}} = \langle \hat{L}_x^2 - \hat{L}_y^2 \rangle, \tag{S14b}$$

$$P'_{\mathrm{lin}} = \langle \hat{L}_x \hat{L}_y + \hat{L}_y \hat{L}_x \rangle. \tag{S14c}$$

Making use of the fact that the total exciton spin $\mathbf{J} = \hat{\mathbf{s}}_{\mathrm{e}} + \hat{\mathbf{s}}_{\mathrm{h}}$ and that the state with $J = 0$ is optically inactive, one can readily check that

$$P_{\mathrm{oo}} = \langle \hat{s}_z^{\mathrm{e}} + \hat{s}_z^{\mathrm{h}} \rangle, \tag{S15a}$$

$$P_{\mathrm{lin}} = 2\langle \hat{s}_x^{\mathrm{e}} \hat{s}_x^{\mathrm{h}} - \hat{s}_y^{\mathrm{e}} \hat{s}_y^{\mathrm{h}} \rangle, \tag{S15b}$$

$$P'_{\mathrm{lin}} = 2\langle \hat{s}_x^{\mathrm{e}} \hat{s}_y^{\mathrm{h}} + \hat{s}_y^{\mathrm{e}} \hat{s}_x^{\mathrm{h}} \rangle. \tag{S15c}$$

Here, to avoid confusion, we use superscipts to denote the type of particle e or h in the exciton and subscripts to denote the Cartesian components.

Equations (S15) clearly demonstrate that to obtain circular polarization of emission, the polarization of electron or hole is sufficient. At the same time, linear polarization requires the correlation of the electron and hole spins: The product of the involved electron and hole spin components should be non-zero meaning that the spins should be correlated. Note that the approach with the exciton spin operator $\hat{\mathbf{L}}$ allows one to derive (in the limiting case of a sufficiently high exchange interaction) the set of coupled equations for the exciton polarization in agreement with Ref. S19. A very similar analysis holds also for the regime of weak exchange interaction where both singlet and triplet states are mixed by the magnetic field, as seen from the general form of Eqs. (S15).

It is important to note that linearly polarized emission by the exciton can be related, in addition to the presence of spin correlations, to entangled electron-hole spin states in the form

$$\Psi_{ent} = \frac{1}{\sqrt{2}}\left[|\uparrow,\Uparrow\rangle + e^{\mathrm{i}\phi}|\downarrow,\Downarrow\rangle\right], \tag{S16}$$

where ϕ describes the orientation of the linear polarization plane. Indeed, the wavefunction (S16) is not factorable into a product of a single electron and a single hole state. From the quantum-mechanical standpoint, measurement of the electron spin $\uparrow$ or $\downarrow$ renders the hole in the spin state $\Uparrow$ or $\Downarrow$, respectively, corresponding to spin entanglement of the two charge carriers. Naturally, the state (S16) corresponds to complete correlation of the electron and hole spins. The first and second product state in Eq. (S16) are the states active in σ^+ and σ^- circular polarizations, respectively (basic states ϕ_1 and ϕ_4 in Eq. (S2a)) and their linear superposition with equal weights corresponds to linear polarization, whose orientation is determined by the phase ϕ. Our theoretical analysis shows, however, that in the limit of either a negligible ($|\Delta_{\mathrm{X}} \ll |g_{\mathrm{V,X}}\mu_B B|$) or a strong ($|\Delta_{\mathrm{X}} \gg |g_{\mathrm{V,X}}\mu_B B|$) exchange interaction, the electron-hole spin state remains a product state in the form

$$\propto (|\uparrow\rangle + e^{\mathrm{i}\alpha_e}|\downarrow\rangle)\otimes(|\Uparrow\rangle + e^{\mathrm{i}\alpha_h}|\Downarrow\rangle),$$

with the phases α_e and α_h. The formation of entanglement for intermediate exchange/magnetic fields, $|\Delta_{\mathrm{X}}| \sim |g_{\mathrm{V,X}}\mu_B B|$, and in presence of anisotropy and its experimental implications are interesting avenues for future research.

Here we mention two possible scenarios of PL generation and exciton recombination (see also concluding paragraphs of Sec. S8 C): excitons that emit light can either be formed (i) from (partially) polarized electrons and holes that retain some degree of polarization during energy relaxation or (ii) from unpolarized resident carriers and polarized photocarriers. The comparison of the linear and circular polarization degrees in Figures 4a and 4c in the main text shows that the number of excitons formed by mechanism (i) is smaller than the number of those from (ii). The process (i) probably corresponds to geminate exciton formation where the electron-hole pair formed by photon absorption binds into an exciton. Further measurements including the degree of linear polarization for linearly polarized excitation (exciton alignment) and comparison of the total emission intensity in case of circular and linear excitation [cf. Ref. S23] may further elucidate the processes, geminate or bimolecular, of exciton formation in perovskite crystals.

It is worthwhile to note that the transient linear polarization memory was studied at room temperature by means of time-resolved differential transmission with linearly polarized pump and probe beams in lead halide perovskite polycrystalline films [S24, S25]. In Ref. [S24] the relatively long dynamics lasting over 150 ps was explained by the crystal anisotropy, e.g., at room temperature detected in tetragonal $MAPbI_3$, but absent in cubic $MAPbI_{1.1}Br_{1.9}$ [S24]. In Ref. [S25] fast dynamics (< 3 ps) were measured for $MAPbI_3$, $MAPbBr_3$ and $CsPbBr_3$ films and related to dynamic structural anisotropies that perturb the local band structure and accessible electronic states. These phenomena are based on mechanisms different from the optical alignment of the exciton spins by means of linearly polarized excitation, as we report in our study here.

C. Hanle effect

The spin dynamics of excitons and resident carriers occur on different time scales. Hence, to analyze the exciton Hanle effect extracted from the dynamics of the degree of optical orientation, it is necessary to develop a suitable theoretical model. In the experiment, we measure I^{++} and I^{+-}, the intensities of the σ^+ and σ^- polarized components of the PL, excited by σ^+ polarized light. To calculate the degree of optical orientation, we used the ratio

$$P_{\mathrm{oo}} = \frac{I^{++} - I^{+-}}{I^{++} + I^{+-}}.$$

The difference of intensities $I^{++} - I^{+-}$ is determined by the spin density of excitons or charge carriers S_z, and the total intensity $I^{++} + I^{+-}$ corresponds to the population $N(t)$.

The Hanle effect results from depolarization of the photoluminescence in a transverse magnetic field applied in the Voigt geometry ($\mathbf{B}_\mathrm{V} \perp \mathbf{k}$). Here, we briefly discuss the contributions of (i) resident electrons and holes and (ii) excitons to the Hanle effect.

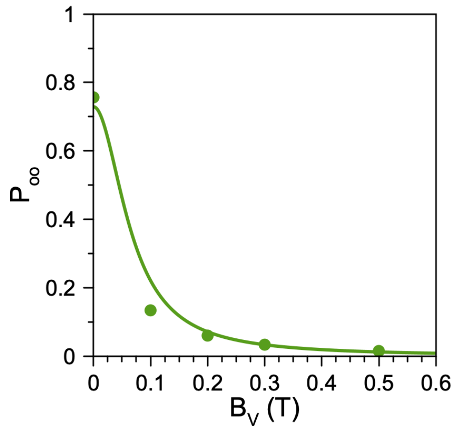

FIG. S7. Hanle effect of exciton depolarization in a transverse magnetic field. P_oo dependence on B_V is shown by the dots measured for σ^+ excitation at $E_\mathrm{exc} = 1.675\,\mathrm{eV}$, $E_\mathrm{det} = 1.508\,\mathrm{eV}$, $P = 30\,\mathrm{mW/cm^2}$ and $T = 1.6\,\mathrm{K}$. The fitting by Eq. (S17) gives the $T_\mathrm{s} = 75\,\mathrm{ps}$ and $\tau_\mathrm{X} = 85\,\mathrm{ps}$.

The contribution of long-living charge carriers to the Hanle effect can be described by the standard expressions, see, e.g., Refs. S26 and S27. For long spin relaxation times exceeding nanoseconds, the electron and hole Hanle curves are extremely narrow: 4 mT for electrons and 18 mT for holes. The free carrier contribution to $P_\mathrm{oo}(B_\mathrm{V})$ is not observed in the range of fields up to a 0.5 T used in the experiment, see Fig. 2f of the main text.

To isolate the exciton contribution, we integrate the measured intensities I^{++} and I^{+-} over time up to $t_1 = 600$ ps which exceeds the exciton lifetime, but is much shorter than the carrier spin relaxation time. In this way, we determine the values $P_\mathrm{oo}(B_\mathrm{V})$ plotted in Figure S7a. To analyze the experimental data, we assume that for the relevant magnetic fields ($B_\mathrm{V} \lesssim 100$ mT) the exchange interaction in the exciton is dominating in this regime. Thus, a simple calculation after Eq. (S11) shows that

$$P_\mathrm{oo}(B_\mathrm{V}) = \frac{\tau_\mathrm{s}}{\tau_\mathrm{X} + \tau_\mathrm{s}} \cdot \frac{P_\mathrm{oo}(t=0)}{1 + (\mu_\mathrm{B} g_\mathrm{V,X} B_\mathrm{V} T_\mathrm{s}/2\hbar)^2}, \tag{S17}$$

where $T_s = \tau_\mathrm{X}\tau_\mathrm{s}/(\tau_\mathrm{X} + \tau_\mathrm{s})$ is the exciton spin lifetime with τ_s being the exciton spin relaxation time, and $P_\mathrm{oo}(0)$ is the optical orientation degree of excitons in zero field. This equation describes the Hanle curve for continuous wave excitation or case of T_s being much shorter than the integration time of the TRPL dynamics. Naturally, the steady-state P_oo at $B_\mathrm{V} = 0$ T is given by $P_\mathrm{oo}(t=0)\tau_\mathrm{s}/(\tau_\mathrm{s} + \tau_\mathrm{X})$.

Fitting the P_oo dependence on B_V with Eq. (S17) gives the half-width at half-maximum $\delta B_\mathrm{X} = 2\hbar/(\mu_\mathrm{B} g_\mathrm{V,X} T_\mathrm{s}) =$ 65 mT and $T_\mathrm{s} = 150$ ps, calculated with $g_\mathrm{V,X} = 2.3$. Note that the estimation of T_s from the Hanle half-width is a rough approximation, since the use of this expression means that the integration time of the experimental signals is much longer than the spin lifetime. The width of the Hanle curve of $\delta B_\mathrm{X} = 65$ mT corresponds to $T_\mathrm{s} = 75$ ps. Using the exciton lifetime $\tau_\mathrm{X} = 85$ ps measured at $P = 30\ \mathrm{mW/cm^2}$, we obtain $\tau_\mathrm{s} = 300$ ps.

Note that the very large value of $P_\mathrm{oo} = 0.6$, which remains constant in the temporal range from 100 to 500 ps (in fact, we have measured this value up to 2 ns), evidences remarkably long spin relaxation times of electrons and holes (at least for one of this carrier, but most probably for both). Using as an estimate for the electron-hole recombination time the lower limit $\tau_\mathrm{R2} = 840$ ps, we obtain $\tau_\mathrm{s,car} \geq 1.5\tau_\mathrm{R2} = 1260$ ps.

S8. CALCULATIONS

A. Band structure calculations

For the calculations, we use the empirical tight-binding (ETB) approach in its nearest-neighbor $sp^3d^5s^*$ version [S28]. It allows us to reproduce the dispersion calculated in state-of-art density functional theory (DFT) with meV precision

for the few bands near the band gap [S29]. For the band structure calculations we relied on the modified Becke-Johnson exchange–correlation potential [S31] in Jishi parametrization [S32] as implemented in the WIEN2k package [S33].

B. Absorption

Let us first remind how the light absorption is calculated as the calculation of the depolarization has a similar structure. The light absorption up to a constant equals to the imaginary part of the dielectric permittivity. The imaginary part of the polarization-averaged dielectric function is given by [S34]

$$\mathrm{Im}\left\{\varepsilon(E)\right\} = \frac{4\pi\alpha\hbar c}{V}\sum_{i,a,\mathbf{k}}\frac{\frac{1}{3}\left|\langle i,\mathbf{k}\left|\mathbf{v}\right|a,\mathbf{k}\rangle\right|^2}{E^2}\delta\left[E-E_a(\mathbf{k})+E_i(\mathbf{k})\right]\,, \tag{S18}$$

where α is the fine structure constant, c is the light velocity, V is the unit cell volume, $\langle i,\mathbf{k}\left|\mathbf{v}\right|a,\mathbf{k}\rangle$ is the matrix element of velocity calculated with the states $|i,\mathbf{k}\rangle$ in the valence and $|a,\mathbf{k}\rangle$ in the conduction bands, $E_i(\mathbf{k})$ and $E_a(\mathbf{k})$ are the energies of the conduction and valence band states with wave vector $\mathbf{k}$. In numerical calculations, the δ-function is approximated with a Gaussian with fixed width. We compute $\langle i,\mathbf{k}\left|\mathbf{v}\right|a,\mathbf{k}\rangle$ in the tight-binding approach using the parameters given in Table S2. We implement the parameters from Ref. S29, fitted to reproduce the band structure obtained in DFT calculations, but change the diagnoal energy of the p-orbital of Pb (E_{pc}) to fit exactly the experimental band gap. We carefully checked that the polarization as a function of energy is not sensitive to such small changes of the band gap. Detailed discussion of comparison ETB with $\mathbf{k}\cdot\mathbf{p}$ model can be found in SI of Refs. S11 and S30. The change of parameter E_{pc} leads to slight change of basic $\mathbf{k}\cdot\mathbf{p}$ parameters which are now: $E_g = 1.52$ eV, $\Delta = 1.262$ eV, $m_h/m_0 = 0.203$, $m_e/m_0 = 0.194$, $g_h = 0.19$, $g_e = 1.33$ (c.f. Table S2 in Ref. S11).

TABLE S2. Tight-binding parameters used in the calculations. In addition to the parameters presented in the table, $s_c s_a^* \sigma$, $s_a^* d_c \sigma$, $p_c d_a \sigma$, $p_a d_c \pi$ and the parameters involving the s_a and s_c^* orbitals are assumed to be zero.

	Ref. S29	expt. corrected
E_{sc}	−5.7767	−5.7767
E_{s^*a}	19.6780	19.6594
E_{pa}	−2.3350	−2.3350
E_{pc}	4.4825	4.6587
E_{da}	10.8491	10.8491
E_{dc}	13.9357	13.9357
$s_c p_a \sigma$	1.0421	1.0421
$s_a^* p_c \sigma$	2.7092	2.7092
$s_c d_a \sigma$	0.3749	0.3749
$pp\sigma$	−1.8838	−1.8838
$pp\pi$	0.1955	0.1955
$p_a d_c \sigma$	1.0341	1.0341
$p_c d_a \pi$	−0.7960	−0.7960
$dd\sigma$	−1.1231	−1.1231
$dd\pi$	2.0000	2.0000
$dd\delta$	−1.4000	−1.4000
$\Delta_a/3$	0.3250	0.3250
$\Delta_c/3$	0.4892	0.4892

The calculated absorption is shown in Figure 5a of the main text. It is in a good agreement with other calculations (see Ref. S35 or more recent Ref. S36). Note that the exciton effects are not included here. They would add a sharp peak right below the gap energy at the R point [S37] and increase the absorption near the M point. It is well known [S35] that the absorption increases significantly from the band gap at the R point up to the M point. Figure 5b shows the calculated band dispersion for bulk $FA_{0.9}Cs_{0.1}PbI_{2.8}Br_{0.2}$ along the $\Gamma\to R\to M\to X$ path.

C. Calculation of optical orientation

We recall that in experiment the circular (or linear) polarization of the emission in the exciton range of the spectrum is measured after non-resonant excitation, typically, well above the free-particle band gap. As a result, free electrons and holes with significant excess kinetic energies are generated, they loose their energy and eventually form excitons

(direct in real space or separated due to potential fluctuations, see Ref. S8 and references therein), before they recombine leading to light emission. Thus, to describe the optical orientation one needs to take into account three basic steps:

1. The circularly polarized optical pulse with energy $E_{\rm exc}$ and wave vector $\mathbf{k}_{\rm exc}$ transfers an electron from the valence band state with wavevector $\mathbf{k}_i$ to the conduction band state with wavevector $\mathbf{k}_a$. satisfying the energy and momentum conservation laws: $E_a(\mathbf{k}_a) - E_i(\mathbf{k}_i) = E_{\rm exc}$; $\mathbf{k}_a - \mathbf{k}_i = \mathbf{k}_{\rm exc}$. The large speed of light allows neglecting the photon wave vector and the momentum conservation law reduces to $\mathbf{k}_a = \mathbf{k}_i \equiv \mathbf{k}_0$ (note that we work in the electron representation here). For a fixed energy of incident light, there is a set of possible $\mathbf{k}_0$ satisfying $E_a(\mathbf{k}_0) - E_i(\mathbf{k}_0) = E_{\rm exc}$, see Figure 5e of the main text for illustration. The spin state of the photoexcited carriers is defined by the polarization of incident light and is described by the density matrix of the carriers $\hat{\rho}^{c,v}(\mathbf{k}_0)$, see below.

2. Electrons and holes loose their kinetic energies, first mostly due to emission of optical phonons, and near the R point due to emission of acoustic phonons. Assuming a definite mechanism of energy relaxation, one may compute the final density matrix of carriers $\hat{\rho}^{c,v}(\mathbf{k}_R)$ in the R point.

3. The electrons and holes recombine at the R point and the polarization of the emitted light may be calculated from $\hat{\rho}^{c,v}(\mathbf{k}_R)$.

Let us now analyze the spin states of the photocarriers in more detail. Let us focus on the conduction electron, the general formalism for the holes is the same. The conduction band Bloch state $|i,\mathbf{k}\rangle$ can be decomposed in the two basic spinors $\uparrow$ and $\downarrow$, corresponding to the free electron spin z component $s_z = +1/2$ and $-1/2$, respectively:

$$|i,\mathbf{k}\rangle = e^{\mathrm{i}\mathbf{k}\cdot\mathbf{r}}\left[U_{i\mathbf{k}}(\mathbf{r})\uparrow + V_{i\mathbf{k}}(\mathbf{r})\downarrow\right]. \tag{S19a}$$

Here the $U_{i\mathbf{k}}(\boldsymbol{r})$, $V_{i\mathbf{k}}(\boldsymbol{r})$ are the periodic Bloch amplitudes. We consider a centrosymmetric phase of the crystal, hence, the counterpart state with the same momentum and the same energy but opposite spin orientation is

$$|\bar{i},\mathbf{k}\rangle = \mathcal{PT}\,|i,\mathbf{k}\rangle = e^{\mathrm{i}\mathbf{k}\cdot\mathbf{r}}\left[U^*_{i-\mathbf{k}}(\mathbf{r})\downarrow - V^*_{i,-\mathbf{k}}(\mathbf{r})\uparrow\right], \tag{S19b}$$

where $\mathcal{P}$ and $\mathcal{T}$ are the space and time reversal symmetry operators. Hereafter, these two conduction band states are denoted by the spin $s = \pm 1/2$ subscript. Similar relations hold for the valence band states and we denote corresponding states in the doublet by the subscript $m = \pm 1/2$.

The general equations (S19) are valid for any $\mathbf{k}$ in the Brillouin zone. In the vicinity of the R point, where the conduction band minimum and the valence band maximum are located, the Bloch functions take the form:

$$\psi^c_{+1/2,R}(\mathbf{r}) = -\frac{\mathcal{Z}(\mathbf{r})}{\sqrt{3}}\uparrow - \frac{\mathcal{X}(\mathbf{r}) + \mathrm{i}\mathcal{Y}(\mathbf{r})}{\sqrt{3}}\downarrow, \tag{S20a}$$

$$\psi^c_{-1/2,R}(\mathbf{r}) = \frac{\mathcal{Z}(\mathbf{r})}{\sqrt{3}}\downarrow - \frac{\mathcal{X}(\mathbf{r}) - \mathrm{i}\mathcal{Y}(\mathbf{r})}{\sqrt{3}}\uparrow, \tag{S20b}$$

for the conduction band and

$$\psi^v_{+1/2,R}(\mathbf{r}) = \mathrm{i}\mathcal{S}(\mathbf{r})\uparrow, \tag{S21a}$$

$$\psi^v_{-1/2,R}(\mathbf{r}) = \mathrm{i}\mathcal{S}(\mathbf{r})\downarrow, \tag{S21b}$$

for the valence band Bloch functions at the R point. The notations $\mathcal{S}(\mathbf{r})$, $\mathcal{X}(\mathbf{r})$, $\mathcal{Y}(\mathbf{r})$, and $\mathcal{Z}(\mathbf{r})$ reflect the symmetry of the corresponding orbital states. The equations (S20) and (S21) are consistent with the irreducible representations $R^{6,\mp}$ relevant for the conduction and valence band in the cubic perovskites [S8, S11].

The density matrix of charge carriers after photoexcitation is given by [S26]:

$$\rho^c_{ss'}(\mathbf{k}_0) = \frac{\sum_{m=\pm1/2}[\mathbf{v}_{sm}(\mathbf{k}_0)\cdot\mathbf{e}][\mathbf{v}_{s'm}(\mathbf{k}_0)\cdot\mathbf{e}]^*}{\sum_{m=\pm1/2,s=\pm1/2}|\mathbf{v}_{sm}(\mathbf{k}_0)\cdot\mathbf{e}|^2}, \tag{S22a}$$

$$\rho^v_{mm'}(\mathbf{k}_0) = \frac{\sum_{s=\pm1/2}[\mathbf{v}_{sm}(\mathbf{k}_0)\cdot\mathbf{e}][\mathbf{v}_{s'm}(\mathbf{k}_0)\cdot\mathbf{e}]^*}{\sum_{m=\pm1/2,s=\pm1/2}|\mathbf{v}_{sm}(\mathbf{k}_0)\cdot\mathbf{e}|^2} \tag{S22b}$$

for the electrons and holes, respectively. Here $\mathbf{e}$ is the light polarization unit vector.

In Eqs. (S22), the basis states of electron and hole are arbitrary. It is convenient to align them along the incident light polarization: For any light polarization γ ($\gamma = +$ for right circular polarization, $\gamma = -$ for left circular polarization)

given by the vector $\mathbf{e}^\gamma$ the matrix elements of the velocity projection onto the polarization vector $\mathbf{v}\cdot\mathbf{e}^\gamma$ are diagonal in the basis found by singular value decomposition (SVD). Its singular values are v_ζ^γ where ζ enumerates the "pure" transitions. At the R point of the Brillouin zone only one of them is non-zero, while both are generally non-zero for a $\mathbf{k}_0$ that does not correspond to any of high symmetry points. Both $\mathbf{v}\cdot\mathbf{e}^+$ and $\mathbf{v}\cdot\mathbf{e}^-$ have the same common singular values and the states that diagonalize them ($v_0^+ = v_1^-$ and $v_1^+ = v_0^-$). In this basis, both density matrices are diagonal and equal. They reduce to

$$\rho^{c+}_{ss'}(\mathbf{k}_0) \equiv \rho^{v+}_{ss'}(\mathbf{k}_0) = \frac{|v_s^+(\mathbf{k}_0)|^2}{|v_0^+(\mathbf{k}_0)|^2 + |v_1^+(\mathbf{k}_0)|^2}\delta_{ss'}\,. \tag{S23}$$

In Eq. (S23) and below, we consider only the right-circular polarization of excitation and omit the superscript $\gamma = +$. We stress that at the R point the selection rules are such that circularly polarized light creates electrons and holes with pure states in the basis of Eqs. (S20), (S21)

$$\sigma^+ \to (+1/2_e, +1/2_h), \quad \sigma^- \to (-1/2_e, -1/2_h). \tag{S24}$$

However, the band mixing at $\mathbf{k} \neq \mathbf{k}_R$ leads to a violation of the selection rules and an effective depolarization of charge carriers [S26, S38].

The most complicated part of the calculation is the description of the evolution of the density matrix during energy relaxation of the carriers. Here we use two simplified approaches: (i) the "effective phonon" model, where we assume that there is a phonon mode which directly transfers electrons and holes from their initial state to the R point of the Brillouin zone, and (ii) the "effective emission" model, where we assume that the polarization of the emission is determined by the selection rules in the excited states. In both cases we neglect excitonic effects, their role is briefly discussed after description of these models below.

1. "Effective phonon" model

In the effective phonon model we assume that there is an "effective" phonon with wavevector $\mathbf{q} = \mathbf{k}_0 - \mathbf{k}_R$ and energy $\hbar\Omega_c(\mathbf{q}) = E_c(\mathbf{k}_0) - E_c(\mathbf{k}_R)$, which provides efficient scattering of the photoelectron from the initial state to the conduction band minimum. A similar phonon with $\hbar\Omega_v(\mathbf{q}) = E_v(\mathbf{k}_R) - E_v(\mathbf{k}_0)$ provides the hole scattering to the R point from the photoexcited state. We assume that the interaction with such "effective" phonons is spin-independent. As a result, the loss of polarization is related to two effects:

1. Violation of the clean selection rules (S24) at high wavevectors of the charge carriers, which are valid only at the R point. It results in spin depolarization of the photoexcited electrons.

2. Mismatch of the spin orientation in the initial state $|i, \mathbf{k}_0\rangle$ and in the final state of a given carrier, Eqs. (S20) for the conduction band and Eqs. (S21) for the valence band in the phonon scattering (Elliott mechanism of spin relaxation, see Refs. S39 and S40 and Sec. S8 C 3 below).

Technically, the density matrix of the states at the R point is found employing the transition matrices

$$V_{0R}^{\eta=c,v} \sim \begin{pmatrix} \left\langle 0_\eta, \mathbf{k}_R \middle| e^{i(\mathbf{k}_R-\mathbf{k}_0)\mathbf{r}} \middle| 0_\eta, \mathbf{k}_0 \right\rangle & \left\langle 0_\eta, \mathbf{k}_R \middle| e^{i(\mathbf{k}_R-\mathbf{k}_0)\mathbf{r}} \middle| 1_\eta, \mathbf{k}_0 \right\rangle \\ \left\langle 1_\eta, \mathbf{k}_R \middle| e^{i(\mathbf{k}_R-\mathbf{k}_0)\mathbf{r}} \middle| 0_\eta, \mathbf{k}_0 \right\rangle & \left\langle 1_\eta, \mathbf{k}_R \middle| e^{i(\mathbf{k}_R-\mathbf{k}_0)\mathbf{r}} \middle| 1_\eta, \mathbf{k}_0 \right\rangle \end{pmatrix} \tag{S25}$$

which allow one to find the density matrix as

$$\rho^\eta(\mathbf{k}_R; \mathbf{k}_0) = V_{0R}^\eta \rho^\eta(\mathbf{k}_0)\,(V_{0R}^\eta)^+ \tag{S26}$$

and compute the intensities of light emitted in the left and right circular polarizations. Given the density matrices $\rho^{c,v}(\mathbf{k})$ for carriers with arbitrary wave vector $\mathbf{k}$, written in the basis of circularly polarized states, the intensity of optical transitions in the right- and left-circular polarization may be calculated as:

$$I^+_{\mathbf{k}}(\mathbf{k}_0) = \rho^c_{00}\rho^v_{00}|v_0(\mathbf{k})|^2 + \rho^c_{11}\rho^v_{11}|v_1(\mathbf{k})|^2 + \rho^c_{01}\rho^v_{10}v_0(\mathbf{k})v_1^*(\mathbf{k}) + \rho^c_{10}\rho^v_{01}v_1(\mathbf{k})v_0^*(\mathbf{k})\,, \tag{S27a}$$

$$I^-_{\mathbf{k}}(\mathbf{k}_0) = \rho^c_{00}\rho^v_{00}|v_1(\mathbf{k})|^2 + \rho^c_{11}\rho^v_{11}|v_0(\mathbf{k})|^2 + \rho^c_{01}\rho^v_{10}v_1(\mathbf{k})v_0^*(\mathbf{k}) + \rho^c_{10}\rho^v_{01}v_0(\mathbf{k})v_1^*(\mathbf{k})\,. \tag{S27b}$$

To calculate the polarization of output light, the intensity of light emitted in the two polarizations should be integrated over the wave vector (c.f. Eq. (S18)):

$$I^+_{\mathbf{k}}(E) = \int_{\mathbf{k}_0} I^+_{\mathbf{k}}(\mathbf{k}_0)\delta\left(E - E_c\left(\mathbf{k}_0\right) + E_v\left(\mathbf{k}_0\right)\right)\,, \tag{S28a}$$

$$I^-_{\mathbf{k}}(E) = \int_{\mathbf{k}_0} I^-_{\mathbf{k}}(\mathbf{k}_0)\delta\left(E - E_c\left(\mathbf{k}_0\right) + E_v\left(\mathbf{k}_0\right)\right)\,. \tag{S28b}$$

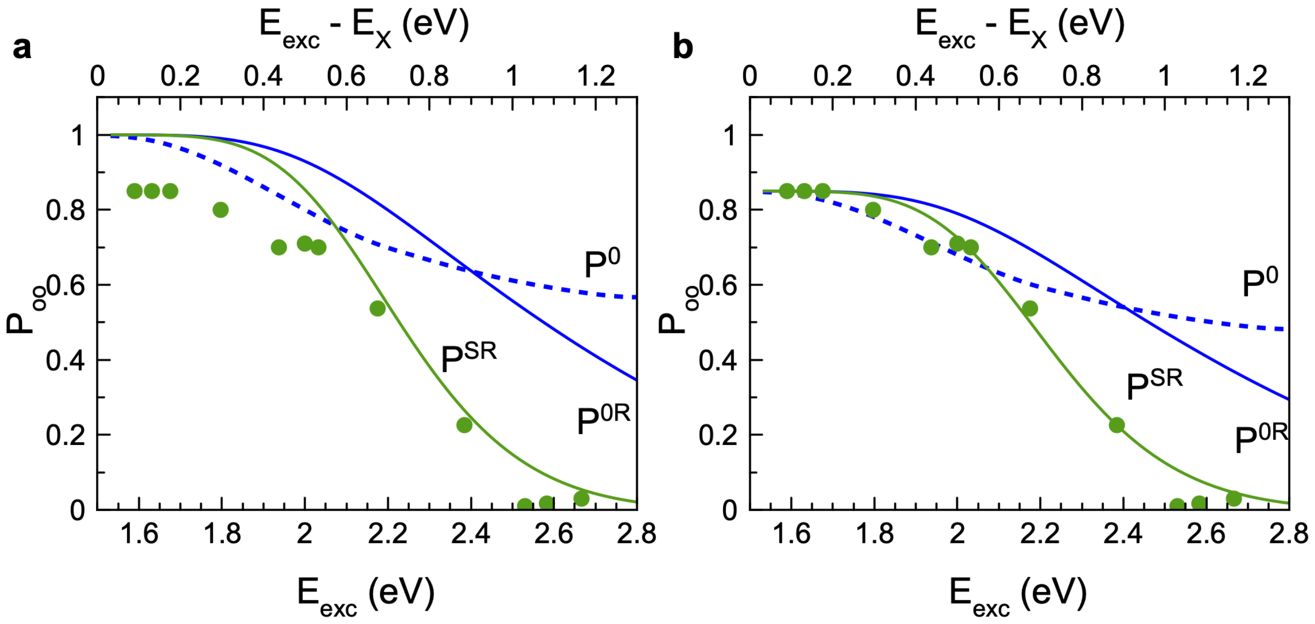

FIG. S8. Theoretically calculated $P^0(E)$, see Eq. (S31) (dashed blue line), $P^{0R}(E)$, see Eq. (S29) (solid blue line) and $P^{SR}(E)$, Eqs. (S27,S28,S38) (solid green line). Points show the experimental values of the optical orientation degree as a function of the detuning energy. Panel (b) shows the same results, but the theory curves are multiplied by the depolarization factor 0.85 to match the experimental value of $P_{oo} = 0.85$ for the small detuning energy of 0.1 eV.

The polarization in this mode is then found from Eqs. (S26-S28) as

$$P^{0R}(E) = \frac{I^+_{\mathbf{k}_R}(E) - I^-_{\mathbf{k}_R}(E)}{I^+_{\mathbf{k}_R}(E) + I^-_{\mathbf{k}_R}(E)}\,. \tag{S29}$$

The results of these calculations are shown in Figure S8 by the solid blue line. All matrix elements in Eqs. (S23,S25) as well as state energies are calculated in the empirical tight-binding approach. In calculations we take a $50 \times 50 \times 50$ k-mesh in 1/8 of the (cubic) Brillouin Zone.

2. "Effective emission" model

In the "effective emission" model we calculate the polarization of emission from the photoexcited states, without an account of the energy relaxation (i.e., the optical orientation of hot excitons). Then, Eq. (S27) with the use of Eq. (S23) reduces to

$$I^+_{\mathbf{k}_0}(\mathbf{k}_0) = \frac{|v_0|^6 + |v_1|^6}{\left(|v_0^+(\mathbf{k}_0)|^2 + |v_1^+(\mathbf{k}_0)|^2\right)^2}\,, \tag{S30a}$$

$$I^-_{\mathbf{k}_0}(\mathbf{k}_0) = \frac{|v_0|^4|v_1|^2 + |v_1|^4|v_0|^2}{\left(|v_0^+(\mathbf{k}_0)|^2 + |v_1^+(\mathbf{k}_0)|^2\right)^2}\,. \tag{S30b}$$

The upper boundary of the optical orientation degree may be calculated from Eqs. (S28,S30) as

$$P^0(E) = \frac{I^+_{\mathbf{k}_0}(E) - I^-_{\mathbf{k}_0}(E)}{I^+_{\mathbf{k}_0}(E) + I^-_{\mathbf{k}_0}(E)}\,. \tag{S31}$$

In the calculations we again take a $50 \times 50 \times 50$ k-mesh in 1/8 of the (cubic) Brillouin Zone. The results of the polarization (S31) as a function of energy are shown in Figure S8 by the dashed blue line. The mesh used for calculating the states and polarization is the same as for the effective phonon model.

3. *Analysis of the results*

Let us discuss briefly the obtained results in the two simplified models plotted by the blue lines in Figure S8. Overall, the behavior of the optical orientation degree as function of detuning found from the model analysis is similar to that observed in experiment: up to a detuning of about 0.5 eV the optical orientation degree is practically constant and for larger detunings it drops. Both theoretical curves (at large detunings) are above the experimental data. This is because additional mechanisms of spin relaxation, namely, the Yafet contribution to the Elliott-Yafet mechanism [S41] and the Bir-Aronov-Pikus mechanism [S42] are disregarded. They may contribute to the spin depolarization of electrons and holes. Let us analyze these effects in more detail.

To that end, we still consider a simplified approach, where the electrons and holes are excited, then loose their excess energy independently, and subsequently bind into excitons before recombination. We also assume that the electron-hole scattering is negligible and disregard the Bir-Aronov-Pikus mechanism of spin relaxation related to the electron-hole exchange [S26, S42], see SI to Ref. S8 for a brief description of the Bir-Aronov-Pikus mechanism in perovskites. We assume that both for electrons and holes their momentum relaxation is fast compared to their spin and energy relaxation. As a result, the spin dynamics of each type of charge carrier can be described by a kinetic equation in the form

$$\frac{d\mathbf{s}(\varepsilon)}{dt} + \frac{\mathbf{s}(\varepsilon)}{\tau_s(\varepsilon)} = Q_\varepsilon\{\mathbf{s}(\varepsilon)\}. \tag{S32}$$

Here ε is the charge carrier energy reckoned from the conduction band bottom (for electrons) or valence band top (for holes), $\mathbf{s}(\varepsilon)$ is the energy-dependent spin distribution function of electrons or holes, $\tau_s(\varepsilon)$ is the energy-dependent spin relaxation time. $Q_\varepsilon\{\mathbf{s}(\varepsilon)\}$ is the phonon-carrier collision integral describing the energy relaxation. Within this simplified approach the additional effects of spin relaxation appear during thermalization of charge carriers [S38]:

$$\mathbf{s}(0) = \zeta(\Delta E)\mathbf{s}(\Delta E), \tag{S33}$$

where

$$\zeta(\Delta E) = \exp(-\Phi), \quad \Phi = \int_{\varepsilon_0}^{\Delta E} \frac{d\varepsilon}{\varepsilon}\frac{\tau_\varepsilon(\varepsilon)}{\tau_s(\varepsilon)}\,. \tag{S34}$$

Here $\tau_\varepsilon(\varepsilon)$ is the energy relaxation time and ε_0 is a constant on the order of the phonon energy ($\varepsilon_0 \ll \Delta E$), for energies smaller than ε_0 the optical phonon emission becomes suppressed by the energy conservation law. The depolarization factor $\zeta(\Delta E)$, where ΔE is the energy of the charge carrier above (below) the band bottom (top), is generally different for electrons and holes.

The depolarization effect described by Eq. (S33) can be quite significant. Let us consider the generic and simplified case, when the detuning energy is much greater than the optical phonon energy, and take into account the scattering via this optical phonon only. In that case the energy relaxation occurs via emission of a cascade of optical phonons. We take the Fröhlich (piezo-optical) mechanism [S43] of the electron-longitudinal optical phonon interaction for illustration. The energy relaxation rate due to the electron-phonon interaction with the phonon mode of energy $\hbar\Omega$ is given by

$$\frac{1}{\tau_\varepsilon(\varepsilon)} = \frac{1}{\tau_{PO}}\left(\frac{\hbar\Omega}{\varepsilon}\right)^{3/2}, \tag{S35}$$

where $\tau_{PO} = (2\alpha\Omega)^{-1}$ is the characteristic electron (or hole) phonon interaction time with α being the Fröhlich constant [S43]. In the simplest model the spin relaxation rate within the Elliott-Yafet mechanism can be written as [S26]

$$\frac{1}{\tau_s(\varepsilon)} = \frac{C(\varepsilon)}{\tau_{PO}}\left(\frac{\hbar\Omega}{\varepsilon}\right)^{1/2}. \tag{S36}$$

Here the function $C(\varepsilon) = \xi(\varepsilon/E_g)^2$ provides the ratio of the spin-flip to the momentum relaxation rates owing to the generic $[\mathbf{k}\times\mathbf{k}']\mathbf{s}$ terms in the electron (hole)-phonon interaction Hamiltonian. ξ is a constant on the order of unity, which depends on the type of the charge carrier and is related to the strength of the spin-orbit coupling (in perovskites the spin-orbit splitting of the conduction band is on the order of the band gap [S11]). The exponent in Eq. (S34) then reads

$$\Phi(\Delta E) = \frac{\xi}{3}\frac{(\Delta E)^3}{\hbar\Omega E_g^2}. \tag{S37}$$

This factor can be quite significant for $\Delta E = E - E_g \sim 1$ eV, resulting in a large depolarization, $\zeta(\Delta E) \to 0$. As a result, inclusion of the spin relaxation processes beyond the effective phonon model can account for the discrepancy between the simplified models above and the experiment. Interestingly, for deformational optical scattering where $\tau_\varepsilon \propto (\hbar\Omega/\varepsilon)^{1/2}$ and $\tau_s \propto C(\varepsilon)(\varepsilon/\hbar\Omega)^{1/2}$ the function $\Phi(\Delta E)$ takes essentially the same form as in Eq. (S37).

To analyze the effect of spin relaxation quantitatively, we include the simplified model presented above in the calculations of the optical orientation by replacing $\rho^\eta(\mathbf{k}_R;\mathbf{k}_0)$ in Eqs. (S27,S28) with the matrix ($n_\eta = \mathrm{Tr}\left\{\rho^\eta(\mathbf{k}_R;\mathbf{k}_0)\right\}$)

$$\rho^{\eta,\mathrm{SR}}(\mathbf{k}_R;\mathbf{k}_0) = n_\eta + \left[\rho^\eta(\mathbf{k}_R;\mathbf{k}_0) - n_\eta\right]\exp\left(-\frac{\xi_\eta}{3}\frac{|E_\eta(\mathbf{k}_0) - E_\eta(\mathbf{k}_R)|}{\hbar\Omega E_g^2}\right). \tag{S38}$$

Then, Eqs. (S27,S28,S38) give an estimate of the optical orientation degree $P^{\mathrm{SR}}(E)$. In Figure S8 we show $P^{\mathrm{SR}}(E)$ by the green line, calculated with $\hbar\Omega = 15$ meV and $\xi_c = \xi_v = 1$. The energy of the phonon mode is chosen based on typical energies of phonons responsible for maximum electron-phonon interaction (see e.g. Ref. S45). The numerical results assume that $P^{\mathrm{SR}}(E)$ only weakly depends on the value of $\hbar\Omega$. While the polarization strongly depends on both values of ξ, we have found surprisingly good agreement between the experimental data and the calculated results for $\xi_c = \xi_v = 1$ without any additional fitting.

Above, we discussed the depolarization of free carriers. The optical orientation degree of the emission is also influenced by the exciton formation processes. Here, two scenarios are possible [cf. Sec. S7 B]:

- Electrons and holes loose their energy independently and then excitons are formed from partially polarized charge carriers. In that case the optical orientation degree equals to the exciton polarization degree and takes the form [S46]

$$P_X = \frac{P_e + P_h}{1 + P_e P_h}, \tag{S39}$$

  where P_e and P_h are the polarization degrees of electrons and holes, respectively, calculated with account for the depolarization factors (S33) and (S37). Note that in this way the polarization of excitons can be higher than the polarization of the individual carriers.

- In the other scenario the partially polarized electrons and holes recombine with unpolarized long-living (resident) carriers. In that case the emission polarization will be given by the average of the electron and hole polarizations, provided a negligible resident carriers' polarization is present.

While the analysis above provides a qualitative and even semi-quantitative description of the optical orientation in perovskites for non-resonant excitation, we abstain from a detailed comparison between the models and the experiments. In fact, the derivation of the depolarization factor (S33) is correct only for not too high excess energies, where the Fröhlich interaction can be described macroscopically and where the spin-orbit contributions to the scattering matrix element take a simple $[\mathbf{k}\times\mathbf{k}']\mathbf{s}$ form. Development of a full theory of spin and energy relaxation in perovskites goes beyond the present work.

[S1] O. Nazarenko, S. Yakunin, V. Morad, I. Cherniukh, M. V. Kovalenko. Single crystals of caesium formamidinium lead halide perovskites: solution growth and gamma dosimetry. *NPG Asia Mater.* **2017**, *9*, e373.

[S2] A. A. Zhumekenov, M. I. Saidaminov, M. A. Haque, E. Alarousu, S. P. Sarmah, B. Murali, I. Dursun, X.-H. Miao, A. L. Abdelhady, T. Wu, O. F. Mohammed, O. M. Bakr. Formamidinium lead halide perovskite crystals with unprecedented long carrier dynamics and diffusion length. *ACS Energy Lett.* **2016**, *1*, 32–37.

[S3] D. P. Mcmeekin, G. Sadoughi, W. Rehman, G. E. Eperon, M. Saliba, M. T. Horäntner, A. Haghighirad, N. Sakai, L. Korte, B. Rech, M. B. Johnston, L. M. Herz, H. J. Snaith. A mixed-cation lead mixed-halide perovskite absorber for tandem solar cells. *Science* **2016**, *351*, 151–155.

[S4] N. J. Jeon, J. H. Noh, W. S. Yang, Y. Ch. Kim, S. Ryu, J. Seo, S. Il Seok. Compositional engineering of perovskite materials for high-performance solar cells. *Nature* **2015**, *517*, 476–480.

[S5] V. M. Goldschmidt. Die Gesetze der Krystallochemie. *Naturwissenschaften* **1926**, *14*, 447–485.

[S6] Zh. Li, M. Yang, J.-S. Park, S.-H. Weit, J. J. Berry, K. Zhu. Stabilizing perovskite structures by tuning tolerance factor: Formation of formamidinium and cesium lead iodide solid-state alloys. *Chemistry of Materials* **2016**, *28*, 284–292.

[S7] P. S. Whitfield, N. Herron, W. E. Guise, K. Page, Y. Q. Cheng, I. Milas, M. K. Crawford. Structures, phase transitions and tricritical behavior of the hybrid perovskite methyl ammonium lead iodide. *Scientific Reports* **2016**, *6*, 35685.

[S8] E. Kirstein, D. R. Yakovlev, M. M. Glazov, E. Evers, E. A. Zhukov, V. V. Belykh, N. E. Kopteva, D. Kudlacik, O. Nazarenko, D. N. Dirin, M. V. Kovalenko, M. Bayer. Lead-dominated hyperfine interaction impacting the carrier spin dynamics in halide perovskites. *Advanced Materials* **2022**, *34*, 2105263.

[S9] V. V. Belykh, D. R. Yakovlev, M. M. Glazov, P. S. Grigoryev, M. Hussain, J. Rautert, D. N. Dirin, M. V. Kovalenko, M. Bayer. Coherent spin dynamics of electrons and holes in $CsPbBr_3$ perovskite crystals. *Nat. Commun.* **2019**, *10*, 673.

[S10] E. Kirstein, D. R. Yakovlev, E. A. Zhukov, J. Höcker, V. Dyakonov, M. Bayer. Spin dynamics of electrons and holes interacting with nuclei in $MAPbI_3$ perovskite single crystals. *ACS Photonics* **2022**, *9*, 1375.

[S11] E. Kirstein, D. R. Yakovlev, M. M. Glazov, E. A. Zhukov, D. Kudlacik, I. V. Kalitukha, V. F. Sapega, G. S. Dimitriev, M. A. Semina, M. O. Nestoklon, E. L. Ivchenko, N. E. Kopteva, D. N. Dirin, O. Nazarenko, M. V. Kovalenko, A. Baumann, J. Höcker, V. Dyakonov, M. Bayer. The Landé factors of electrons and holes in lead halide perovskites: universal dependence on the band gap. *Nat. Commun.* **2022**, *13*, 3062.

[S12] A. D. Wright, R. L. Milot, G. E. Eperon, H. J. Snaith, M.B Johnston, L.M. Herz. Band-tail recombination in hybrid lead iodide perovskite. *Adv. Funct. Mater.* **2017**, *27*, 1700860.

[S13] V. S. Chirvony, J. P. Martínez-Pastor. Trap-limited dynamics of excited carriers and interpretation of the photoluminescence decay kinetics in metal halide perovskites. *J. Phys. Chem. Lett.* **2018**, *9*, 4955–4962.

[S14] D. W. deQuilettes, K. Frohna, D. Emin, T. Kirchartz, V. Bulovic, D. S. Ginger, S. D. Stranks, Charge-carrier recombination in halide perovskites. *Chem. Rev.* **2019**, *119*, 11007—11019.

[S15] A. Bercegol, F. J. Ramos, A. Rebai, T. Guillemot, D. Ory, J. Rousset, V. Lombez. Slow diffusion and long lifetime in metal halide perovskites for photovoltaics. *J. Phys. Chem. C* **2018**, *122*, 24570–24577.

[S16] D. Kudlacik, N. E. Kopteva, M. Kotur, D. R. Yakovlev, K. V. Kavokin, C. Harkort, M. Karzel, E. A. Zhukov, E. Evers, V. V. Belykh, M. Bayer. Optical spin orientation of localized electrons and holes interacting with nuclei in an $FA_{0.9}Cs_{0.1}PbI_{2.8}Br_{0.2}$ perovskite crystal. *arXiv2404.05369* **2024**.

[S17] W. Tao, Q. Zhou, H. Zhu. Dynamic polaronic screening for anomalous exciton spin relaxation in two-dimensional lead halide perovskites. *Sci. Adv.* **6**, *eabb7132*, 2020.

[S18] G. Yumoto, F. Sekiguchi, R. Hashimoto, T. Nakamura, A. Wakamiya, and Y. Kanemitsu. Rapidly expanding spin-polarized exciton halo in a two-dimensional halide perovskite at room temperature *Sci. Adv.* **8**, *eabp8135*, 2022.

[S19] E. L. Ivchenko, G. E. Pikus. Optical orientation and polarized luminescence of excitons in semiconductors, in *Excitons*, ed. by E. I. Rashba and M. D. Sturge, North-Holland Publishing Company, **1982**.

[S20] P. Odenthal, W. Talmadge, N. Gundlach, R. Wang, C. Zhang, D. Sun, Z.-G. Yu, Z. Valy Vardeny, and Y. S. Li. Spin-polarized exciton quantum beating in hybrid organic–inorganic perovskites. *Nature Physics* **2017**, *13*, 894.

[S21] P. Tamarat, M. I. Bodnarchuk, J.-B. Trebbia, R. Erni, M. V. Kovalenko, J. Even, and B. Lounis. The ground exciton state of formamidinium lead bromide perovskite nanocrystals is a singlet dark state. *Nature Materials* **2019**,*18*, 717.

[S22] P. Tamarat, E. Prin, Y. Berezovska, A. Moskalenko, T. P. T. Nguyen, C. Xia, L. Hou, J.-B. Trebbia, M. Zacharias, L. Pedesseau, C. Katan, M. I. Bodnarchuk, M. V. Kovalenko, J. Even, and B. Lounis. Universal scaling laws for charge-carrier interactions with quantum confinement in lead-halide perovskites. *Nature Communications* **2023**, *14*, 229.

[S23] T. Amand, B. Dareys, B. Baylac, X. Marie, J. Barrau, M. Brousseau, D. J. Dunstan, and R. Planel. Exciton formation and hole-spin relaxation in intrinsic quantum wells. *Phys. Rev. B* **1994**, *50*, 11624.

[S24] C. X. Sheng, C. Zhang, Y. Zhai, K. Mielczarek, W. Wang, W. Ma, A. Zakhidov, and Z. V. Vardeny. Exciton versus free carrier photogeneration in organometal trihalide perovskites probed by broadband ultrafast polarization memory dynamics. *Phys. Rev. Lett.* **2015**, *114*, 116601.

[S25] J. P. H. Rivett, L. Z. Tan, M. B. Price, S. A. Bourelle, N. J. L. K. Davis, J. Xiao, Y. Zou, R. Middleton, B. Sun, A. M. Rappe, D. Credgington, and F. Deschler. Long-lived polarization memory in the electronic states of lead-halide perovskites from local structural dynamics. *Nat. Commun.* **2018**, *9*, 3531.

[S26] F. Meier and B. Zakharchenya, eds., *Optical Orientation*, (Horth-Holland, Amsterdam, **1984**).

[S27] M. Glazov. *Electron & Nuclear Spin Dynamics in Semiconductor Nanostructures*, Series on Semiconductor Science and Technology. OUP Oxford, **2018**.

[S28] J.-M. Jancu, R. Scholz, F. Beltram, and F. Bassani. Empirical spds* tight-binding calculation for cubic semiconductors: General method and material parameters. *Phys. Rev. B* **1998**, *57*, 6493.

[S29] M. O. Nestoklon. Tight-binding description of inorganic lead halide perovskites in cubic phase. *Comp. Mat. Science* **2021**, *196*, 110535.

[S30] M. O. Nestoklon, E. Kirstein, D. R. Yakovlev, E. A. Zhukov, M. M. Glazov, M. A. Semina, E. L. Ivchenko, E. V. Kolobkova, M. S. Kuznetsova, and M. Bayer. Tailoring the Electron and Hole Landé Factors in Lead Halide Perovskite Nanocrystals by Quantum Confinement and Halide Exchange. *Nano Lett.* **2023**, *23*, 8218.

[S31] F. Tran and P. Blaha. Accurate band gaps of semiconductors and insulators with a semilocal exchange-correlation potential. *Phys. Rev. Lett.* **2009**, *102*, 226401.

[S32] R. A. Jishi, O. B. Ta, A. A. Sharif. Modeling of lead halide perovskites for photovoltaic applications. *J. Phys. Chem. C* **2014**, *118*, 28344.

[S33] P. Blaha, K. Schwarz, F. Tran, R. Laskowski, G. K. H. Madsen, L. D. Marks. WIEN2k: An APW+lo program for calculating the properties of solids. *J. Chem. Phys.* **2020**, *152*, 074101.

[S34] M. Graf and P. Vogl. Electromagnetic fields and dielectric response in empirical tight-binding theory. *Phys. Rev. B* **1995**, *51*, 4940.

[S35] S. Boyer-Richard, C. Katan, B. Traoré, R. Scholz, J.-M. Jancu, J. Even. Symmetry-based tight binding modeling of halide perovskite semiconductors. *J. Phys. Chem. Lett.* **2016**, *7*, 3833–3840.

[S36] W. Song, G.-Y. Guo, S. Huang, L. Yang, and L. Yang. First-principles studies of second-order nonlinear optical properties of organic-inorganic hybrid halide perovskites. *Phys. Rev. Applied* **2020**, *13*, 014052.

[S37] Sh. Singh, Ch. Li, F. Panzer, K. L. Narasimhan, A. Graeser, T. P. Gujar, A. Köhler, M. Thelakkat, S. Huettner, D. Kabra. Effect of thermal and structural disorder on the electronic structure of hybrid perovskite semiconductor $CH_3NH_3PbI_3$.

J. Phys. Chem. Lett. **2016**, *7*, 3014–3021.
[S38] M. Dyakonov and V. Perel. Spin orientation of electrons associated with interband absorption of light in semiconductors. *Sov. Phys. JETP* **1971**, *33*, 1053.
[S39] R. J. Elliott. Theory of the effect of spin-orbit coupling on magnetic resonance in some semiconductors. *Phys. Rev.* **1954**, *96*, 266.
[S40] Z. Liu, M. Nestoklon, J. Cheng, E. Ivchenko, and M. Wu. Spin-dependent intravalley and intervalley electron-phonon scatterings in germanium. *Physics of the Solid State* **2013**, *55*, 1619.
[S41] Y. Yafet. *g*-factors and spin-lattice relaxation of conduction electrons. In F. Seitz and D. Turnbull, editors, *Solid State Physics*, page 2. (Academic, New-York, **1963**).
[S42] G. L. Bir, A. G. Aronov, and G. E. Pikus. Spin relaxation of electrons due to scattering by holes. *Sov. Phys. JETP* **1975**, *42*, 705.
[S43] V. F. Gantmakher and Y. B. Levinson. *Carrier Scattering in Metals and Semiconductors.* (North-Holland Publishing Company, **1987**).
[S44] A. D. Wright, C. Verdi, R. L. Milot, G. E. Eperon, M. A. Pérez-Osorio, H. J. Snaith, F. Giustino, M. B. Johnston, and L. M. Herz. Electron–phonon coupling in hybrid lead halide perovskites. *Nature Communications* **2016**, *7*, 11755.
[S45] S. Poncé, M. Schlipf, and F. Giustino. Origin of low carrier mobilities in halide perovskites. *ACS Energy Lett.* **2019**, *4*, 456.
[S46] V. P. Kochereshko, E. L. Ivchenko, D. R. Yakovlev, and P. Lavallard. Resonant optical orientation and alignment of excitons in superlattices. *Phys. Solid State* **1998**, *40*, 2024.
